# Coupled nickel and molybdenum isotopes quantify the evolution of the ocean system since the Late Cretaceous

**DOI:** 10.1126/sciadv.aeg0341

**Published:** 2026-08-26

**Authors:** Mingzhao Sun, Corey Archer, Florian Scholz, Tim Sweere, Derek Vance

**Affiliations:** ^1^ Institute of Geochemistry and Petrology, Department of Earth Sciences, ETH Zürich, 8092 Zürich, Switzerland.; ^2^ Department of Earth System Sciences, Universität Hamburg, Bundesstraße 55, 20146 Hamburg, Germany.

## Abstract

Nickel isotopes (δ^60^Ni) are an emerging tool for understanding ocean biogeochemical evolution, given improved modern Ni budget constraints. Here, we provide constraints on past ocean Ni isotope compositions from organic-rich sediments from Tarfaya Basin core SN°4. The data constrain Late Cretaceous ocean δ^60^Ni to ∼+0.8 to +1.0 per mil (‰), lower than the modern value of +1.34‰. Mass balance modeling suggests two drivers of this difference: changes in riverine input isotope composition during the Cenozoic and a more reducing Late Cretaceous ocean. Because Ni and Mo isotopes respond differently to changes in ocean inputs and redox-sensitive sinks, coupled Ni-Mo models help to deconvolve the relative importance of the two processes. The results show that the areal fraction of the euxinic sink was higher than today, reaching ∼2% during OAE2 (Oceanic Anoxic Event 2), and that the importance of the deep ocean oxic sink relative to the euxinic sink increased during the Cenozoic, potentially driven by changes in the biological pump and ocean circulation.

## INTRODUCTION

The stepwise oxygenation of the ocean and atmosphere, from anoxic conditions in the Archean to mostly oxygenated conditions in the later Phanerozoic, was associated with key evolutionary events such as the appearance of photosynthesis, eukaryotes, and animals (*1*, *2*). Even after atmospheric O_2_ levels reached close to modern levels, expansions of marine anoxia have been convincingly linked to major biotic crises, including the “big five” mass extinctions of the Palaeozoic and Mesozoic (*3*, *4*). These intervals, which were often associated with perturbations to the carbon cycle, highlight the importance of marine anoxia as one of the key stressors for life (*5*, *6*). Studies of the interplay between ocean redox, climate, and the biosphere rely on our ability to accurately reconstruct past environmental conditions. However, existing geochemical tracers come with considerable uncertainties (*7*), limiting detailed assessments of causal relationships, especially further back in Earth history. Moreover, only a few tracers are capable of reconstructing the global ocean state (*8*–*10*), for example, the global prevalence of bottom water anoxia in response to the main drivers of surface Earth redox, rather than local redox conditions shaped by paleogeographic contingencies that can produce anoxia in partially isolated marginal basins even in the modern well-oxygenated oceans. Last, most well-established tracers, such as the Mo and U isotope systems (*8*, *9*), typically distinguish best between states like the modern oxygenated ocean and strongly reducing conditions, broadly including anoxic and euxinic conditions, whereas intermediate situations are less easily identified.

Recent advances in our understanding of the modern ocean Ni isotope budget (*11*–*19*) suggest that this novel isotope system can provide complementary information to established proxies. Not only might Ni isotopes provide more nuanced constraints on intermediate redox levels, but its application may also be more sensitive to the isotope composition of the input fluxes. Many established tracers of ocean redox rely on an isotopic mass balance that partition the (modern) input flux into different redox-controlled sedimentary sinks (*8*, *9*, *20*, *21*). These approaches rarely consider the potential for confounding secular variability in the isotope composition of the input flux itself. This complexity is potentially tractable using multiple tracers that show variable sensitivities to both the nature of the sinks and the isotope composition of the inputs to the ocean.

Nickel is a bioessential trace metal involved in key enzymatic processes (*22*, *23*), but it also exhibits redox-sensitive behavior through its association with metal oxides and organic matter (*12*, *16*, *24*–*26*). Recent observations in the modern ocean suggest that this behavior may be used to reconstruct past ocean redox conditions. Mn oxide burial exerts strong leverage on the Ni isotope budget as it is the largest sink and is associated with significant isotope fractionation (*14*, *16*, *17*, *27*). The second largest sink, authigenic Ni burial in sediments of highly productive upwelling margins, is not associated with significant isotope fractionation and can thus provide an archive of the seawater Ni isotope composition (*12*, *15*, *28*).

The dissolved Ni isotope composition of the modern deep ocean is +1.34 ± 0.06 per mil (‰) (mean and SD for observations beneath 500 m) (*18*, *29*). The offset toward heavier isotopes of seawater than the estimated modern input of +0.79‰ (*30*) is ascribed to Ni isotope fractionation during output to the Mn oxide sink. Mass balance calculations yield a δ^60^Ni of +0.42‰ for the total oxic sink, which is consistent with a mixture of Ni output to Mn-rich abyssal and proximal hydrothermal sediments (*16*, *17*, *31*, *32*) (see the Supplementary Materials for additional details of Ni budget in the modern ocean). By contrast, the modern euxinic Ni sink is small and cannot account for the offset between inputs and seawater δ^60^Ni, although the δ^60^Ni value of the euxinic sink is relatively low at +0.45 ± 0.15‰ (*11*).

These features of the modern ocean Ni isotope budget highlight the potential of Ni isotopes as a tracer for past environmental conditions. Here, we use a case study from the Late Cretaceous to test and apply this emerging tracer. We also show how the coupling of Ni and Mo isotopes via mutually consistent isotope budgets quantifies the evolution of the ocean system. This approach not only enables reconstructions of global ocean redox conditions but also provides new insights into changes in the oceanic inputs.

### Background to OAE2 and the MCE

The Cretaceous Period was generally warm, with elevated atmospheric, surface and deep ocean temperatures (*33*–*35*). One of the most extreme carbon cycle perturbations of the Mesozoic, the Cenomanian-Turonian Oceanic Anoxic Event [C-T OAE or OAE2; ∼93.9 million years ago (Ma)], developed under these Late Cretaceous greenhouse conditions. OAE2 is characterized by a pronounced positive carbon isotope excursion linked to enhanced burial of marine organic carbon and expansion of low-oxygen marine environments (*3*). The event lasted ∼400 to 900 thousand years (kyr) (*36*) and is generally considered to be triggered by large-scale magmatic activity (*37*). The Plenus Cold Event (PCE) represents a short-lived global re-oxygenation and cooling phase during the early stages of OAE2 (*38*, *39*). OAE2 is preceded by a prominent short-term event, also associated with a carbon isotope excursion in the middle Cenomanian. This event has been termed the mid-Cenomanian Event (MCE; ∼96.6 Ma) (*40*, *41*).

Sedimentary elemental concentrations and isotope compositions have been widely used to reconstruct environmental changes during OAE2, with a major focus on the global spatial extent of oceanic deoxygenation, as illustrated by studies based on Cr isotopes (*42*), Cd and Zn isotopes (*43*), Tl isotopes (*44*), and V isotopes (*45*). Earth system models have suggested that as much as 50% of the global ocean may have been anoxic during OAE2 (*46*, *47*), broadly consistent with estimates inferred from Tl and Zn isotopes (*44*), but sulfur and other trace metal isotope studies generally indicate a much smaller extent. The most intensely studied trace metal isotope systems, uranium (U) and molybdenum (Mo), both with long residence times in the modern ocean, suggest that anoxic/euxinic conditions covered a maximum of ∼15% of the total seafloor (*9*, *20*, *48*, *49*). These findings are generally consistent with estimates from sulfur isotopes, which suggest that ∼2.5 to 5% of the global seafloor was covered by euxinic waters—roughly 15 to 30 times the modern value of 0.15% (*50*). Acknowledging this significant progress, it is also clear that reconstructions based on single isotope systems come with inherent uncertainties, often related to a poorly constrained isotope fractionation between the aqueous phase of the water column and target sedimentary archives, as well as through local element cycling in the studied settings (*21*, *51*).

## RESULTS

Nickel isotope data for the studied core (SN°4, Tarfaya basin; see fig. S10 for location) are presented in table S1. Elemental concentration data reported for the same core in Sun *et al.* (*52*), as well as other published data (*21*, *53*, *54*), suggest that these sediments were deposited in an open-ocean upwelling setting. This observation suggests that the authigenic Ni isotope compositions of core SN°4 should reflect past ocean compositions (*12*, *15*, *28*).

**Fig. 1.**
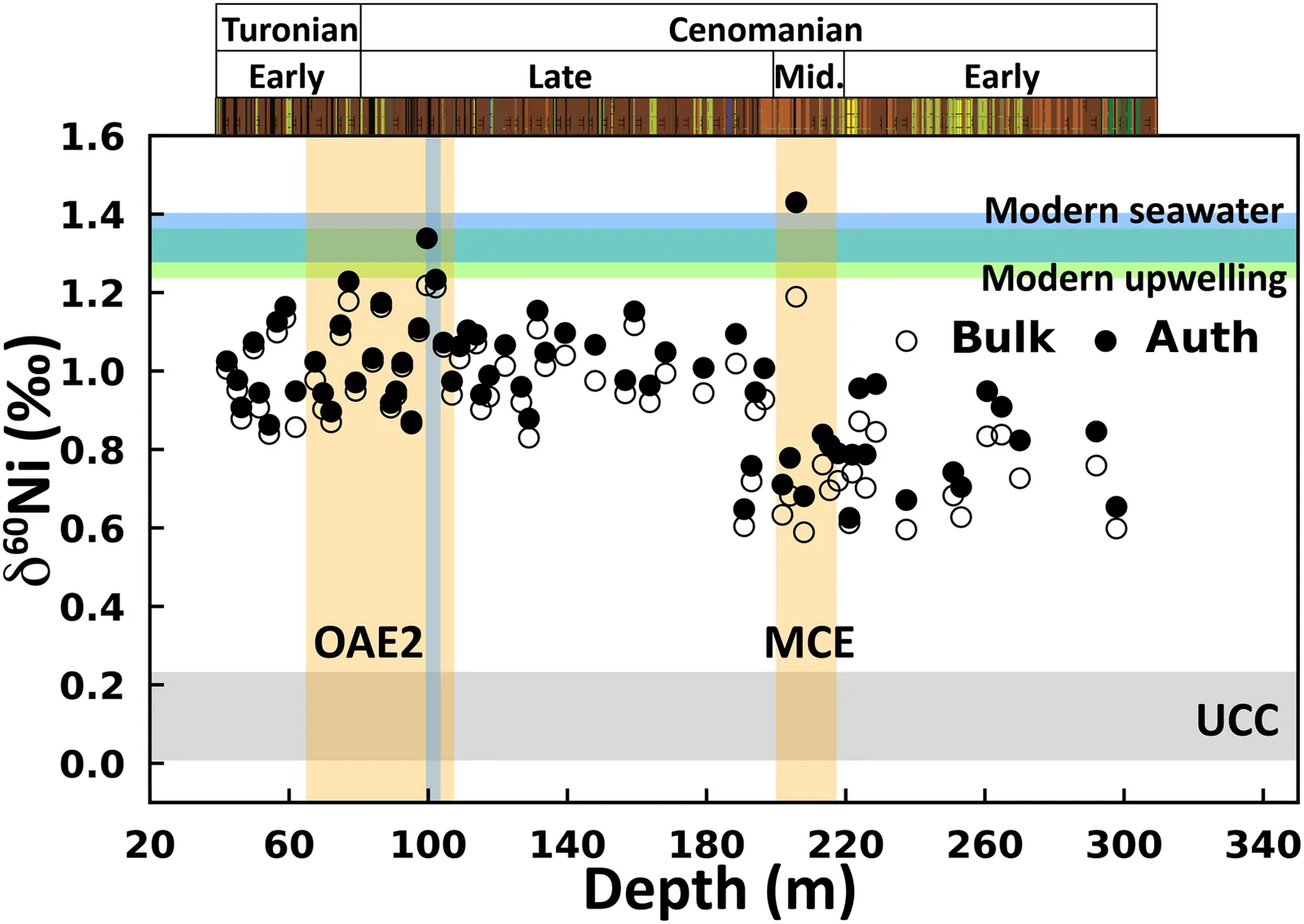
Bulk (black open circles) and authigenic (black filled circles) Ni isotope compositions of core SN°4. The horizontal blue bar shows the Ni isotope compositions of the modern deep ocean [+1.34 ± 0.06‰, mean and SD; data for depths >500 m and as compiled by Lemaitre *et al.* (*29*) with the addition of data from Bian *et al.* (*18*)]. The horizontal green bar shows the Ni isotope composition of organic-rich sediments from the modern Peru and Namibia upwelling margins [+1.30 ± 0.06‰; compiled in (*28*), with the range mostly determined by uncertainty in the Ni/Al_detrital_ ratio used for the detrital correction]. The horizontal gray bar shows the best estimate for the Ni isotope composition of the UCC (+0.12 ± 0.11‰) (*55*, *56*). The lithological legend follows Beil *et al.* (*53*). Black indicates black shales, brown indicates marlstone, and green indicates limestone.

The measured bulk Ni isotope composition must be corrected for detrital or lithogenic contributions to obtain that of authigenic Ni. As in previous studies from this group (*12*, *15*, *28*), the detrital contribution to bulk sample Ni is obtained using the aluminum (Al) content of the sample and an assumed (Ni/Al)_detrital_. The Ni isotope composition of the authigenic fraction is then obtained assuming the detrital fraction has the δ^60^Ni of the upper continental crust (UCC), estimated at 0.12 ± 0.11 (*55*, *56*). Uncertainties in (Ni/Al)_detrital_ propagate to the final [Ni]_authigenic_ and, together with uncertainties in δ^60^Ni_detrital_, further propagate to the calculated authigenic δ^60^Ni. Böning *et al.* (*57*) use the intercept of Ni/Al ratio versus TOC for modern upwelling sediments to define the Ni/Al ratio of the detrital fraction, suggesting values of 0 to 3.3 × 10^−4^, with a best estimate of 1.7 × 10^−4^. Sweere *et al.* (*28*) further noted that using 3.3 × 10^−4^ overestimates the contribution of lithogenic Ni to modern upwelling margin sediments. Here, we adopt a (Ni/Al)_detrital_ of 1.7 × 10^−4^ to correct for the detrital contribution. In any case, the detrital correction is barely significant for all samples in core SN°4, except for one toward the top of the MCE that has a low Ni/Al ratio (Fig. 1) that is not considered further. With a (Ni/Al)_detrital_ ratio of 1.7 × 10^−4^, 93 ± 5% (mean and SD) of total Ni is found to be authigenic, and the correction shifts δ^60^Ni by only 0.05 ± 0.03‰ (mean and SD). The higher Ni/Al ratio of 3.3 × 10^−4^ leads to a further shift of 0.06 ± 0.04‰ relative to the baseline correction using a (Ni/Al)_detrital_ of 1.7 × 10^-4^.

The dominant feature of the authigenic δ^60^Ni record is the generally lower δ^60^Ni values than the modern deep ocean (+1.34 ± 0.06‰; Fig. 1). Only one PCE sample falls in the modern deep ocean range at +1.34. Before and during the MCE, authigenic δ^60^Ni averages +0.79 ± 0.10‰ (mean and SD, *n* = 19), followed by a prominent step up to values +1.01 ± 0.11‰ (mean and SD, *n* = 46, excluding two PCE samples).

## DISCUSSION

In the modern ocean, organic-rich sediments deposited in the cores of the oxygen minimum zones of open ocean upwelling settings record the deep ocean Ni isotope composition (Fig. 1) (*12*, *15*, *28*). Nickel in these sediments is transferred to the sediment in organic matter that is produced in the water column (*12*, *26*). The organic-associated Ni that is released to porewaters by respiration is quantitatively sequestered by porewater sulfide to a solid phase (*16*, *28*), so that the sediment preserves the Ni isotope composition of the overlying water column. Sediments deposited beneath these modern OMZ settings contain little authigenic Mn (*58*–*60*), and bottom waters do not see persistent high levels of sulfide (*60*, *61*). Thus, there is no significant contribution to isotopically light sedimentary Ni from Mn oxides, nor is there significant remobilization in deep sulfidic seawater of isotopically light Ni from Mn oxide particulates (Mn oxide particulate shuttle; c.f. Ni isotopes in the Black Sea water column) (*11*).

Data for multiple trace metals from the same samples studied here (*52*) suggest that the Tarfaya Basin core SN°4 was deposited in an open ocean upwelling margin setting, very similar to the oxygen minimum zones of the modern productive coastal upwelling systems of Peru and Namibia (*21*, *53*, *54*, *62*). These data present no evidence for persistent high levels of free H_2_S in bottom waters nor for strong hydrographic restriction. Furthermore, the Mo_EF_-U_EF_ data for core SN°4 indicate the absence of a particulate shuttle in the Tarfaya Basin (*20*, *49*, *52*), a conclusion further supported by the sedimentary Re/Mo ratio proxy (*52*). Therefore, we interpret the authigenic Ni isotope compositions of the organic-rich sediments from core SN°4 as a record of contemporary global seawater δ^60^Ni.

### Controls on the δ^60^Ni of Late Cretaceous seawater

The observed differences between the modern and the reconstructed Late Cretaceous ocean Ni isotope composition must reflect a significant difference in the Earth surface Ni cycle compared to modern. To assess whether this difference reflects changes in the isotope composition of oceanic inputs, in the balance of the different sinks, or in both, we apply a simple mass balance model. In this model, the δ^60^Ni of the ocean is set by the relative proportions of the different outputs and the isotope composition of the oceanic input. The details of the parameters required are given in the Supplementary Materials. The relevant isotope mass balance equation is

$$\begin{array}{c} \delta_{\text{input}}=f_{\text{oxic}}\;\left(\delta_{\text{sw}}+\Delta_{\text{oxic}}\right)+f_{\text{upwelling}}\;\left(\delta_{\text{sw}}+\Delta_{\text{upwelling}}\right)+\\ f_{\text{euxinic}}\;\left(\delta_{\text{sw}}+\Delta_{\text{euxinic}}\right) \end{array}$$ (1)

where *f*_oxic,upwelling,euxinic_ are the proportional sizes of the three sinks of Ni from the oceanic dissolved pool, Δ_oxic,upwelling,euxinic_ are their isotope fractionations from seawater, and δ_input_ and δ_sw_ are the δ^60^Ni values of the input flux and seawater, respectively. Given the multiple variables involved, a parameter *k* is also introduced, defined as the ratio of the fractional size of the oxic sink to the combined open ocean sinks (upwelling and oxic; Eq. 2) (*8*)

$$k=\frac{f_{\text{oxic}}}{f_{\text{oxic}}+f_{\text{upwelling}}}$$ (2)

**Fig. 2.**
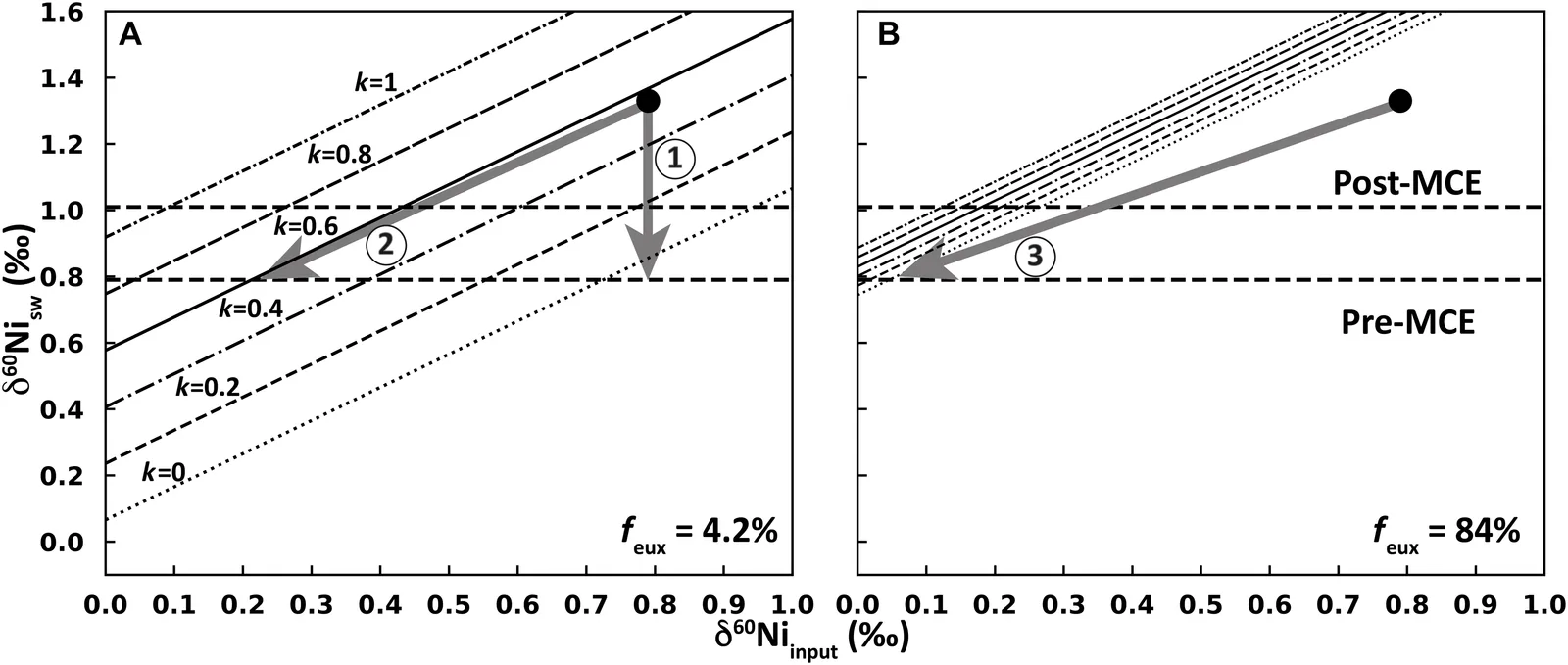
Potential controls of the input flux on the δ^60^Ni of Late Cretaceous seawater. Oceanic Ni isotope composition in the Late Cretaceous as a function of the δ^60^Ni of the input flux for different configurations of the output fluxes (different *k* values and for two different fractional sizes of the euxinic sink of Ni). The black dots represent the modern Ni state. The lower and upper horizontal dashed lines represent the pre-MCE and post-MCE seawater δ^60^Ni, which are +0.79 and +1.01‰, respectively. (**A**) For a euxinic sink that is the same fractional size as in the modern ocean (4.2%; see Table 1). (**B**) For a euxinic sink equivalent to a fractional area ∼20 times larger than the modern ocean [roughly consistent with constraints from sulfur isotopes (*50*)]. Arrows 1 to 3 show how Late Cretaceous oceanic Ni isotope compositions can be explained by changes in the sizes of the sinks or changes in the isotopic composition of the input flux of Ni to the ocean, relative to the modern ocean (see text for further discussion).

Figure 2 illustrates how the oceanic Ni isotope composition changes as a function of the δ^60^Ni of the input flux and different configurations of the output fluxes.

Applying this framework to the Late Cretaceous record, the relatively low δ^60^Ni values (∼+0.8‰) before and during the MCE can be explained by either a lighter isotope composition of the input flux or a smaller proportion of isotopically light Ni burial. The calculation in Fig. 2A, for a fractional euxinic sink similar to today, suggests that the Ni isotope data for the Late Cretaceous could be explained by an input flux δ^60^Ni close to modern rivers, but only if the oxic sink was almost entirely absent (*k* = 0 to 0.2; arrow 1 on Fig. 2A). An oxic sink similar in size to today (*k* = 0.56; Table 1) would require an input δ^60^Ni value much closer to the UCC, at ∼+0.25‰ for the pre-MCE period or at +0.45‰ for the post-MCE (arrow 2 on Fig. 2A). For either period, a fractional euxinic area of the oceans substantially greater than modern, within the range of 15 to 30 times larger as inferred from sulfur isotopes (*50*) (here illustrated by a representative value of ∼20 times), would require a δ^60^Ni_input_ much lower than today (see arrow 3 on Fig. 2B), although even then the pre-MCE data can barely be reproduced.

**Table 1.** Mutually consistent Ni-Mo budget for the modern ocean.

Best estimate (Observation)				MC simulation Mean ± 1 SD (median)*			
Inputs				Inputs			
Ni (10^8^ mol/year)†	3.68			Ni (10^8^ mol/year)	3.68 ± 0.37 (3.68)		
δ^60^Ni (‰)†	0.79			δ^60^Ni (‰)	0.79 ± 0.10 (0.79)		
Mo (10^8^ mol/year)‡	3.30			Mo (10^8^ mol/year)	3.22 ± 0.17 (3.22)		
δ^98^Mo (‰)‡	0.70			δ^98^Mo (‰)	0.69 ± 0.07 (0.69)		
**Outputs**	**Oxic**	**Upwelling**	**Euxinic**	**Outputs**	**Oxic**	**Upwelling**	**Euxinic**
Oceanic area (10^14^ m^2^)	3.6						
Fractional area (%)	90	1.1	0.08		90	1.1	0.08
Ni sink (10^8^ mol/year)	1.98§	1.53¶	0.17#	Ni sink (10^8^ mol/year)	1.98 ± 0.38 (1.98)	1.54 ± 0.06 (1.54)	0.16 ± 0.07 (0.16)
Fractions (%)	53.8	41.6	4.6	Fractions (%)	53.8 (53.8)	42.0 (41.9)	4.2 (4.4)
δ^60^Ni (‰)	0.42§	1.30¶	0.45#	δ^60^Ni (‰)	0.41 ± 0.22 (0.41)	1.30 ± 0.06 (1.30)	0.45 ± 0.15 (0.45)
Ni acc. rate (μmol/m^2^/year)**	0.61	38.6	59.0	Ni acc. rate (μmol/m^2^/year)**	0.61 (0.61)	39.0 (39.0)	54.2 (55.7)
Mo sink (10^8^ mol/year)††	1.40	1.70	0.20	Mo sink (10^8^ mol/year)	1.39 ± 0.13 (1.39)	1.63 ± 0.09 (1.63)	0.20 ± 0.02 (0.20)
Fractions (%)	42.3	51.6	6.1	Fractions (%)	43.2 (43.2)	50.6 (50.6)	6.2 (6.2)
δ^98^Mo (‰)††	−0.61	1.64	1.84	δ^98^Mo (‰)	−0.60 ± 0.09 (−0.60)	1.65 ± 0.09 (1.65)	1.84 ± 0.10 (1.84)
Mo acc. rate (μmol/m^2^ per year)**	0.43	43.0	69.4	Mo acc. rate (μmol/m^2^ per year)**	0.43 (0.43)	41.1 (41.2)	69.9 (69.8)
Required Ni/Mo	1.42	0.90	0.85	Modeled Ni/Mo	1.43 ± 0.31 (1.42)	0.95 ± 0.05 (0.94)	0.78 ± 0.37 (0.80)
Ni/Mo‡‡		0.97 ± 0.09	1.1 ± 0.36				
Ni/Mo§§			0.38 ± 0.80				

* All modeled values are reported as the mean ± 1 SD (median).

† Ciscato *et al.* (*12*).

‡ Archer and Vance (*97*) and Kendall *et al.* (*98*).

§ Calculated based on the steady-state Ni mass balance.

¶ Ciscato *et al.* (*12*), He *et al.* (*15*), and Sweere *et al.* (*28*).

# Vance *et al.* (*11*).

** Calculated by dividing the flux by the area.

†† See the Oceanic Mo mass balance section in the Supplementary Materials.

‡‡ From Ni/TOC and Mo/TOC.

§§ From authigenic Ni/Mo.

Lithium isotope data for marine carbonates across OAE2 not only require a large change in the size of the riverine input to the oceans but also a shift to lighter isotope compositions, caused by a move to high weathering intensity on the continents (*63*). Consistent with an increase in Li input flux (*63*), it has been proposed that the Ni input flux during OAE2 was around three times the modern value, given the tight relationship between sedimentary Ni concentration and TOC, the relatively constant Ni/TOC ratios close to the modern value, and the excess organic carbon burial rate (*52*). A similar change in the Ni isotope composition of the input flux is plausible, given the variability of Ni isotopes seen in Amazonian rivers that mirrors those for Li isotopes (*55*, *64*). Specifically, in the modern Amazon basin, rivers draining the rapidly eroding and colder Andean highlands (e.g., Solimoes and Madeira: low weathering intensity, a weathering-limited regime, and hence more incongruent weathering) have δ^60^Ni up to about +1.2 to +1.4‰, whereas rivers that drain the hot and tectonically inactive Amazonian lowlands (e.g., Negro: high weathering intensity, a transport-limited regime, and hence more congruent weathering) have δ^60^Ni much closer to the rocks of the UCC, at +0.2 to +0.4‰ (*55*). Molybdenum also shows this behavior in the Amazon catchment (*65*). Mo isotope compositions of rivers (expressed as per mil deviation from the NIST SRM 3134 value of +0.25‰) (*66*) are as high as ∼+1.5‰ under low weathering intensity but approach the UCC value of about +0.3‰ under high weathering intensity (*65*).

On the other hand, the temporal pattern of global seawater δ^60^Ni differs markedly from that of δ^7^Li recorded in carbonate across OAE2. The Li isotope drop occurs during the OAE2 itself and is restricted to it, with the pre- and post-OAE2 values lying close to those of modern carbonates (*63*). In contrast, the Late Cretaceous Ni isotope shift occurs at the end of the MCE, with little change across OAE2. This apparent absence of coupled changes in the Li and Ni isotope composition of the input could be related to the precise nature of the secondary mineral phases that control their sequestration in soils and the riverine isotope composition, with light Ni preferentially bound to Fe-Mn oxides (*67*, *68*) versus light Li to clay minerals (*64*). These two reservoirs could respond differently to changes in the weathering regime during the carbon cycle perturbations of the Late Cretaceous.

Clearly, the multiple controls on the Ni mass balance rule out its quantitative use to assess both the possibility of changes in weathering regime and its impact on the isotope composition of the input flux and changes in ocean redox. Given that this conclusion should also be considered in applications of other metal isotope systems, we now explore how an approach that combines two metal isotope systems, Ni and Mo, can add further constraints.

### Coupled Ni-Mo isotope modeling of Late Cretaceous redox conditions

Molybdenum isotopes are one of the best established geochemical tracers for past global ocean redox conditions (*8*, *69*–*71*). Because of differences in the isotope budgets, the proxy provides a complementary redox assessment to δ^60^Ni. Specifically, Mo isotopes show large contrasts between the oxic and euxinic sinks, as well as a significant isotope fractionation from bottom water into sediments of the upwelling sink. In contrast, Ni isotopes exhibit similar compositions in oxic and euxinic sinks, with negligible fractionation from the deep water column into sediments of the upwelling sink. This contrast causes the two isotope systems to respond differently to shifts in the ocean’s redox state, allowing for a more robust reconstruction through the combination of the two in a mutually consistent model.

**Fig. 3.**
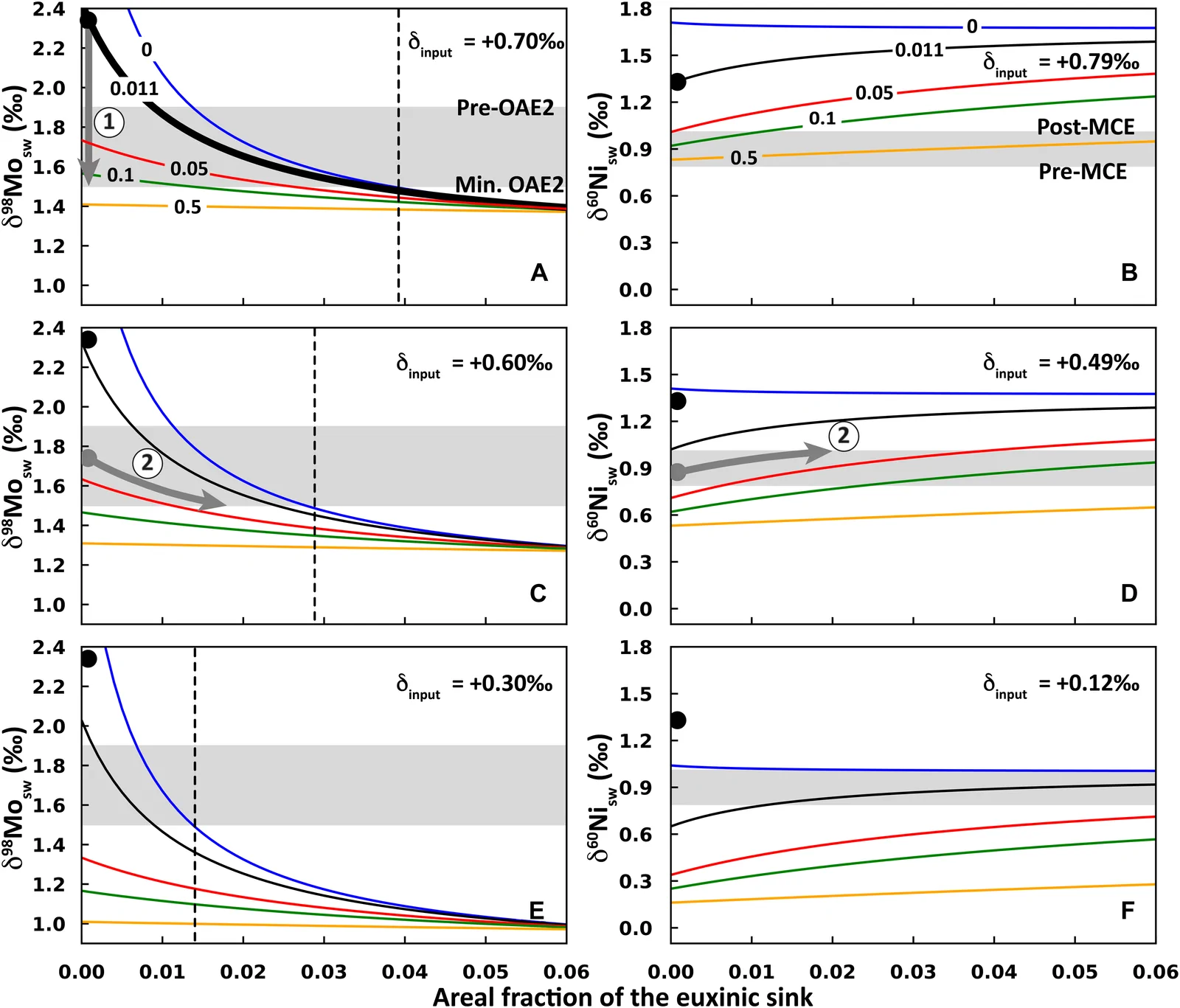
Potential controls of the output flux on the δ^60^Ni of Late Cretaceous seawater. Modeled Mo (**A**, **C**, and **E**) and Ni (**B**, **D**, and **F**) isotope compositions for the Late Cretaceous ocean as a function of the areal fraction of the euxinic sink for different isotope compositions of the input (modern at the top, UCC at the bottom, and for the middle panels, a value halfway between the modern input and the minimum modern Amazon Basin value) and the areal fractions of the upwelling sink (labeled curves). The black dots represent modern states of Mo and Ni, respectively. In the Mo panels on the left, the gray bar shows estimated oceanic δ^98^Mo for the pre-OAE2 period (+1.9‰) and the minimum for the OAE interval itself (+1.5‰). Seawater δ^60^Ni of the pre-MCE and post-MCE are +0.79 and +1.01‰, respectively. See text for references to arrows 1 and 2. The vertical dashed lines indicate the permissible euxinic fractional area for any upwelling fractional area under a fixed Mo isotope composition of the input fluxes.

A number of studies have previously used measurements of organic-rich sediments, with the oceanic mass balance for Mo isotopes, to seek to quantify the balance of sinks in the Late Cretaceous generally and OAE2 in particular (*20*, *21*, *48*, *49*). The interpretation of sedimentary δ^98^Mo values measured in organic-rich sediments from upwelling settings such as Tarfaya in terms of contemporary seawater is complicated by uncertainties concerning the local redox setting and its impact on how δ^98^Mo is recorded in the sediments (*21*, *71*). However, a growing consensus from previous studies of multiple sections across different ocean basins suggests that maximum Late Cretaceous seawater δ^98^Mo values before OAE2 were around +1.90‰, lighter than the modern global seawater value of +2.34‰, and that seawater δ^98^Mo decreased by up to ∼0.4‰ during the OAE (*21*, *48*). Therefore, this consensus range was used to represent the δ^98^Mo of Late Cretaceous seawater (Fig. 3).

Here, we apply a coupled Ni-Mo isotope mass balance model to reconstruct Late Cretaceous conditions, assuming steady-state Ni and Mo cycles. To account for uncertainties in individual parameters in modern Ni and Mo budgets, as well as the uncertainties in the mutual consistency of these two budgets, we performed a Monte Carlo (MC) simulation to identify mutually consistent Ni and Mo budgets (see the Supplementary Materials for details). The modeled mean fluxes and isotope compositions for Ni and Mo (Table 1) were used for their respective marine budgets in Fig. 3. Specifically, Fig. 3 investigates how the oceanic isotope compositions of Mo and Ni change as a function of changes in the fractional areas of the three sinks, given mutually consistent Mo and Ni accumulation rates in those sinks as derived from modern data (see the Supplementary Materials for details). Most modeled scenarios in Fig. 3 result in a total output that is larger than the modern input. However, the relative sizes of the input and outputs only control the size of the oceanic reservoir and the metal concentrations. If removal to each sink is assumed to show a pseudo-first-order dependence on aqueous concentration, the larger or smaller oceanic reservoirs will result in outputs that differ in terms of the magnitude of elemental output fluxes from the oceans, but the isotope balance will not change (*8*). This is because seawater Ni and Mo isotope compositions, the target of interest here, depend on the isotope compositions of the input fluxes, isotope fractionation between seawater and each sink, and the fractional contribution of each sink to the total output flux (Eq. 1), rather than on the absolute magnitude of the output fluxes.

In addition to the potential control of the input flux on the δ^60^Ni of Late Cretaceous seawater (Fig. 2), we use the coupled Ni-Mo isotope mass balance model to explore the role of the output flux while also investigating potential shifts in the isotope compositions of the input flux relative to modern values. Figure 3 shows how δ^98^Mo_sw_ and δ^60^Ni_sw_ (*y* axis) in the Late Cretaceous change as a function of the areal fraction of the euxinic sink (*x* axis) for different areal fractions of the upwelling sink (labeled curves) and three illustrative input isotope compositions: (i) the modern ocean input (top panel); (ii) input compositions equal to the UCC composition, which would reflect an extreme weathering intensity scenario (bottom panel); and (iii) an intermediate state (middle panel) that seeks to understand the impact of a higher weathering intensity regime, where the input lies half way between the modern global flux and the minimum values observed in rivers draining the high weathering intensity catchments of the modern Amazon Basin (*55*, *65*). This intermediate case provides an illustrative sensitivity test between the modern inputs and an extreme congruent-weathering endmember, rather than a uniquely constrained reconstruction of the Late Cretaceous inputs.

There are a number of ways to reproduce δ^98^Mo_sw_ in the Late Cretaceous, both before and during OAE2. For a modern δ^98^Mo input (Fig. 3A), the estimated pre-OAE2 δ^98^Mo_sw_ (+1.9‰) can be achieved with an areal fraction of the euxinic sink of about 0.01, given a modern-like fractional area of the upwelling sink (black curve in Fig. 3A). Alternatively, the same δ^98^Mo_sw_ can be achieved by increasing the fractional area of the upwelling sink to about 0.03 while keeping the euxinic area the same as its modern value (arrow 1 in Fig. 3A). To explain the minimum δ^98^Mo_sw_ during OAE2 (+1.5‰), the fractional area of euxinic seafloor must either increase further to 0.04 (further down the black curve in Fig. 3A) or the upwelling sink must increase to around 0.2. These conclusions remain qualitatively the same for other Mo isotope compositions of the input (Fig. 3, C and E). Quantitatively, however, the closer the Mo isotope composition approaches the UCC the lower the euxinic area permitted by the Mo mass balance (vertical dashed lines on Fig. 3, A, C, and E). For example, for an UCC-like Mo isotope input the OAE2 minimum δ^98^Mo_sw_ requires a fractional area of euxinia smaller than 0.02 for any size of the upwelling sink (Fig. 3E).

Unlike Mo, Ni isotopes are relatively insensitive to the size of the euxinic sink as increases are compensated by decreases in the size of the oxic sink and both sinks have very similar Ni isotope compositions. The pre-MCE δ^60^Ni_sw_ value of +0.79‰ is the same as the modern input and cannot be reproduced by any combination of outputs (Fig. 3B). Thus, an additional constraint that comes from Ni isotopes seems to be that the isotope compositions of the input fluxes have to change from the modern values. Given the likely reason for such a change (weathering regime) and the coupled responses of Ni and Mo isotopes to weathering regime in the modern Amazon Basin, the isotope compositions of the Ni and Mo inputs are likely to shift in tandem. Such potential changes in the input flux have generally been treated as being of secondary importance in Mo mass balance studies (*8*, *21*, *49*).

The Mo and Ni isotope compositions of modern rivers are almost universally heavier than the UCC, so that this latter represents a likely minimum for the inputs to the oceans in the past. The implications of such inputs for the oceanic mass balance are explored in the bottom row of Fig. 3 (E and F). For Ni, Late Cretaceous oceanic isotope compositions can only be explained in terms of a near-zero fractional area of the upwelling sink unless that of the euxinic sink is at the higher end of the range plotted (Fig. 3F). However, such large fractional euxinic areas are precluded by the fact that they result in seawater Mo that is far too light (Fig. 3E).

Thus, together, Mo and Ni isotopes appear to require input fluxes to the late Cretaceous ocean that are intermediate between modern rivers and the UCC. We therefore focus on the middle panels of Fig. 3 (C and D) to assess the degree to which the isotope budgets of the two elements can be reconciled by such inputs. Overall, Late Cretaceous δ^98^Mo_sw_ and δ^60^Ni_sw_ are close to reproduced for low fractional areas of euxinia, probably similar to modern, and fractional area of the upwelling sink of around 0.02 to 0.03 (gray circles on Fig. 3, C and D), with Mo favoring the lower and Ni the higher end of this range depending on the choice made regarding the isotope composition of the input. The minimum estimate for oceanic δ^98^Mo during OAE2 of +1.5‰ and the OAE2 δ^60^Ni value of around +1.0‰ would require an increase in the size of the euxinic sink to about 2% of seafloor area (arrow 2 heading right from gray circles on Fig. 3, C and D). Our estimate is broadly consistent with those inferred from other single isotope systems (*9*, *20*, *50*) but is substantially lower than estimates based on Tl and Zn isotopes (*43*, *44*). Subsequent improvements to the modern marine Zn budget (*72*) may alter the extent of deoxygenation inferred by Sweere *et al.* (*43*), whereas the Tl-based estimate (*44*) is sensitive to the assumption of constant isotope compositions of input fluxes across OAE2 and the isotope compositions of oxic sinks. In summary, coupled Ni-Mo mass balance models provide a more comprehensive framework for constraining the evolution of ancient ocean systems as they not only better quantify past changes in outputs from the ocean but also provide an additional dimension for understanding changes in inputs to the ocean and thus potential shifts in the weathering regime.

### Broader implications: The evolution to modern ocean chemistry since the Late Cretaceous

Late Cretaceous seawater δ^98^Mo and δ^60^Ni patterns, as reconstructed from the Tarfaya Basin core SN°4, published data, and mass balance models of Ni and Mo isotopes, require the δ^98^Mo and δ^60^Ni values of the input fluxes to be at least slightly different from the modern. This model constraint on the Ni-Mo input fluxes in the Late Cretaceous relative to modern, derived from our analysis, is consistent with the suggestion that the weatherability of the continental crust (that is, the reactivity at Earth’s surface) increased through the Cenozoic (*73*) and with increasingly heavy seawater Li isotopes through that interval (*74*).

**Fig. 4.**
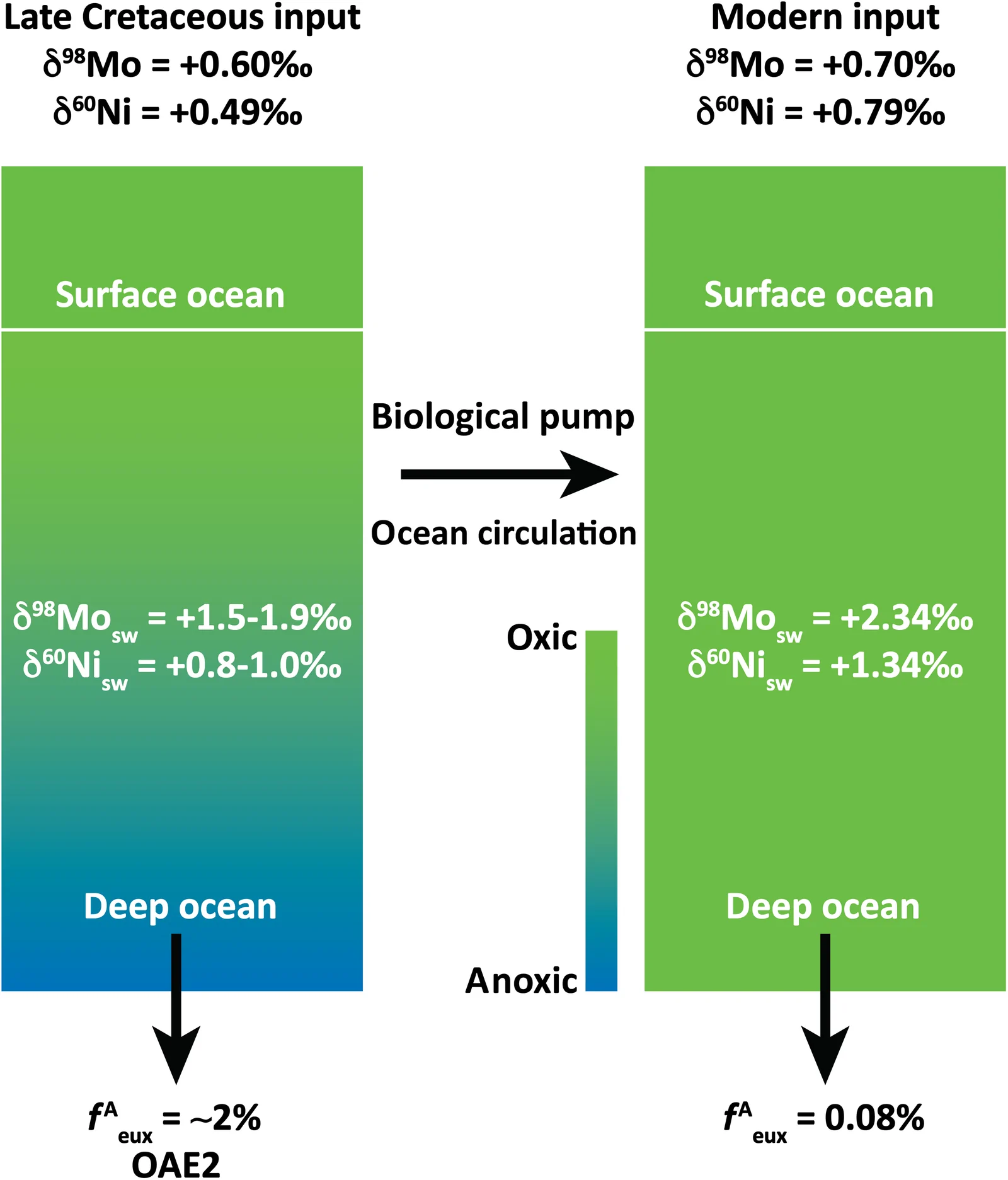
Schematic comparison between inferred Late Cretaceous and modern ocean redox states. The Ni and Mo isotope compositions of input fluxes in the Late Cretaceous are taken from Fig. 3 (C and D). *f*^A^_eux_ denotes the areal fraction of the euxinic sink.

Superimposed on this change in the inputs are changes in the sinks from the oceanic dissolved pool as controlled by ocean redox (Fig. 4). Our data and modeling suggest an areal fraction of the euxinic sink during OAE2 of ∼2% relative to the roughly 0.08% of the modern ocean (Table 1; see the Supplementary Materials for details), broadly consistent with Mo and S isotope studies (*20*, *50*). Both Mo and Ni isotopes also suggest an increase from the background Late Cretaceous state to Cenozoic and modern values: from around +1.0 to +1.34‰ for δ^60^Ni_sw_ and from +1.9 to +2.34‰ for δ^98^Mo_sw_ (*71*, *75*). The sink of both metals to Mn oxide–rich sediments is the dominant driver of the seawater isotope composition of both these metals, given their significantly lighter isotope compositions relative to global seawater and the large proportion of output fluxes to oxic sinks (see the Supplementary Materials for details). The isotopic difference between the modern global riverine flux and the UCC is smaller for δ^98^Mo than for δ^60^Ni (0.4‰ for Mo versus ∼0.7‰ for Ni). Furthermore, the difference between modern seawater and the modern global riverine flux is larger for δ^98^Mo than for δ^60^Ni (∼1.6‰ for Mo versus ∼0.5‰ for Ni). For these reasons, the shift in seawater Mo isotopes is less sensitive to changes in the isotope composition of the input flux (*8*) compared to Ni (Fig. 3). However, the overall agreement between the conclusions from the Ni and Mo budgets adds weight to the suggestion that the oceans was not at modern-like oxygenation levels in the Late Cretaceous (*21*, *71*).

This change in ocean redox could, in principle, have been driven by a change in atmospheric O_2_ levels, with burial of organic matter and pyrite over geological time scales being the only significant long-term source of O_2_ to the atmosphere (*76*, *77*). Reconstructed atmospheric O_2_ evolution since the Late Cretaceous, based on either modeling approaches or geochemical proxies, does not show sufficient temporal resolution to indicate a substantial increase in atmospheric O_2_ after OAE2 (*78*–*81*). Most of these reconstructions suggest that atmospheric O_2_ has differed by less than 5% between the Late Cretaceous and the present day (∼21%). Furthermore, the excess oxygen produced by enhanced burial of reduced organic matter and pyrite during OAE2 itself could have been largely buffered by the oxidation of reductants derived from large igneous provinces (LIPs) (*82*). Therefore, it is unlikely that increasing atmospheric O_2_ was the main driver of differences between the Late Cretaceous and modern ocean redox state, implying additional intraoceanic mechanisms.

Substantial changes in plankton assemblages in the marine ecosystem may have contributed to the oxygenation of the deep ocean from the Late Cretaceous to the modern. In the Mesozoic, the rise in marine eukaryotic phytoplankton diversity was fueled by the changes in sea level, water column stratification, and nutrient levels (*83*). During the Late Cretaceous, eukaryotic phytoplankton—including diatoms, dinoflagellates, and coccolithophores, the three dominant groups of eukaryotic phytoplankton in the modern ocean—began to dominate marine ecosystems (*84*–*86*). The faster sinking of these large, silicified, and calcified primary producers would result in reduced O_2_ consumption in the water column (*87*), thereby perhaps contributing to observed differences between the Late Cretaceous and modern ocean redox conditions (*88*).

Another plausible mechanism involves reorganizations of oceanic circulation patterns. The combination of increased ocean overturn and mixing with a stronger latitudinal temperature gradient across the Cretaceous-Cenozoic boundary may have led to the development of a thermohaline circulation system similar to that of the modern oceans (*89*). This would have increased the ventilation of the deep ocean and led to more oxygenated intermediate and deep waters (*89*). Such reorganizations of oceanic circulation patterns across the Cretaceous-Cenozoic boundary may have been facilitated by the progressive opening of the Central Atlantic Gateway, which connected the South and North Atlantic Oceans (*90*, *91*), promoting the formation of North Atlantic Deep Water (*92*).

Together, the coupled Ni-Mo isotope mass balance model suggests that the Late Cretaceous differed substantially from modern ocean conditions in both redox state and ocean inputs. The results are consistent with a larger relative contribution from oxic sinks relative to euxinic in the modern ocean, likely driven by internal biological and physical changes within the marine system rather than direct atmospheric O_2_ forcing.

## MATERIALS AND METHODS

The samples studied here are from drill core SN°4 from the central part of the Tarfaya Basin in southern Morocco (fig. S1). Around 700 m of Upper Cretaceous laminated biogenic sediments was deposited in the basin, consisting mainly of calcareous nanoplankton, marine organic matter, planktonic foraminifers, and dispersed biogenic silica [e.g., see (*53*, *62*) and references therein]. The lithological variations in core SN°4, shown in Fig. 1, mainly reflect the relative importance of organic matter, carbonate, and terrigenous components (*53*). In this study, we have specifically targeted horizons with high total organic carbon. Tarfaya Basin core SN°4 has been interpreted as an open ocean upwelling margin, very similar to the modern productive coastal upwelling systems [e.g., see (*52*) and references therein].

Bulk sediment samples were digested with a 2:2:3 mixture of concentrated HF, HNO_3_, and HClO_4_ on a hot plate (*54*). Aliquots of this solution were taken for multielement analysis. The methods for the chemical isolation and Ni isotope analysis have been described in detail by Sun *et al.* (*93*), and only the most important aspects are described briefly here.

### Column chromatography

Two different Ni column chromatography procedures were used in this study, the first described in detail by Sun *et al.* (*93*), and the other briefly described here. The first step in this second procedure involves the separation of an impure Ni fraction from other metal elements such as Cu, Fe, Zn, and Cd, using the anion exchange resin, AG MP-1M (Bio-Rad) (*94*). This was followed by a second anion column involving the same resin in a custom-made shrink-fit PTFE column, with a resin bed of 2 cm long by 2 mm in inner diameter. The sample was loaded onto the column in 0.2 M HF after conditioning, and Ni was isolated from Al and Ti via elution with 0.5 ml 0.2 M HF (*95*). A final column step used the chelating resin Nobias PA-1 (Hitachi) and the elution steps as described in the first Ni column chromatography procedure. However, two further elution steps were added between removal of most major cations with a pH-buffered 0.03 M ammonium acetate solution and the final Ni elution with 1 M HCl. The first was that of 10 ml of 2 M NH_4_F at pH 5, used to elute residual Fe, Ti, and Al after matrix removal (*96*). The second involved 2 ml of deionized high-purity water (resistivity = 18.2 MΩ) to remove F^−^. Further details of the new Ni column chromatography elution steps are given in table S2.

### Mass spectrometric measurements

Nickel isotope compositions were measured on a Thermo Fisher Scientific Neptune Plus MC-ICP-MS at ETH Zürich and are reported in the usual delta notation, as per mil deviations from the NIST SRM986

δNi60 (‰)=[(Ni60/Ni58)sample(Ni60/Ni58)NIST SRM986−1]×1000 (3)

The long-term reproducibility of Ni isotope analyses in our lab is 0.07 to 0.08‰ through repeat measurements of primary NIST standards as well as two secondary standards for Ni [USGS Fe-Mn nodules, Nod-P1 (+0.35 ± 0.08‰, 2 sigma, *n* = 808 over 10 years) and Nod-A1 (+1.04 ± 0.07‰, 2 sigma, *n* = 610 over 10 years), bulk digested and passed through the Ni column chemistry]. Where relevant, the diagrams show the uncertainty as represented by the long-term reproducibility, unless the internal precision is larger, in which case the latter is shown.

## Supplementary Materials

### This PDF file includes:

Supplementary Text Figs. S1 to S10 Tables S1 and S2 References
