# Supplementary material for: Coupled nickel and molybdenum isotopes quantify the evolution of the ocean system since the Late Cretaceous
Source: Sci Adv. 2026 Aug 26;12(35):eaeg0341. doi: 10.1126/sciadv.aeg0341 (PMC13510601; doi:10.1126/sciadv.aeg0341)
Supplement: Supplementary file 1 [file sciadv.aeg0341_sm.pdf]

Science Advances
AAAS

Supplementary Materials for

Coupled nickel and molybdenum isotopes quantify the evolution of the ocean system since the Late Cretaceous

Mingzhao Sun *et al.*

Corresponding author: Mingzhao Sun, sunming@stanford.edu

Sci. Adv. **12**, eaeg0341 (2026)
DOI: 10.1126/sciadv.aeg0341

This PDF file includes:

Supplementary Text
Figs. S1 to S10
Tables S1 and S2
References

Supplementary Text

Coupled Ni and Mo mass balance model

In the main paper, we apply a coupled and self-consistent model of the modern mass balances of Ni and Mo and their isotopes to Late Cretaceous sediments, in order to understand ocean redox evolution. Here we describe this model, including how it aligns with our current understanding of organic carbon and Mn burial across different depositional settings. This coupled framework permits improved constraints on the potential changes in inputs and outputs to and from the ocean, and thereby on the biogeochemical and redox evolution of the ancient ocean - beyond just the Late Cretaceous Ocean. In the following we first outline the results of previous studies that have documented the sizes and isotope compositions of the three main sedimentary sinks for Ni and Mo. We then assess the mutual consistency of these budgets by compiling Ni/Mo ratios in the sediments that represent each sink, and their relationships to what we know about global burial of the main carrier or associated phases (organic carbon and Mn oxide), comparing the results to those required by the global mass balances. Finally, we undertake a Monte Carlo simulation to better understand the uncertainties involved in these multiple constraints, and the degree to which a central best-estimate can be derived.

Different terminologies, based on varying criteria, have been used to describe the aqueous redox conditions of depositional settings in both modern and ancient oceans *(99)*, leading to the use of different classifications for the oceanic sinks of redox-sensitive trace metals. Here, the global Ni and Mo output fluxes are partitioned into three main sinks representing three different redox settings, termed the **upwelling, euxinic and oxic sinks**. The specific definitions and representative modern settings of each sink are described below. We note that, though the terminology varies somewhat in previous publications, our scheme essentially follows that used for Mo and its isotopes in Dahl et al. *(100)*, Chen et al. *(8)* and Kendall et al. *(73)*.

Upwelling margin sediments in open ocean systems are generally characterized by high total organic carbon (TOC) content due to high primary productivity in the photic zone of the water column above, and are often enriched in authigenic trace metals. This type of open ocean upwelling margin is here referred to as the **upwelling sink**, typified in the modern ocean by the Peru and Namibian Margins. This setting is characterized in the modern ocean by a pronounced oxygen minimum zone (OMZ) *(101)*. Bottom waters are typically low in dissolved oxygen. Dissolved H_2S is restricted to sediment porewater *(8, 60, 102)* but can be intermittently released into the overlying water column during sulfidic events *(103)*. A number of different terms have been used in previous studies to refer to the processes encapsulated in this "upwelling sink", sometimes with ambiguous and inconsistent definitions. The upwelling setting described here is conceptually equivalent to the suboxic sink of Scott et al. *(104)*, the reducing sink of Poulson Brucker et al. *(105)* and Reinhard et al. *(102)*, the weakly oxic sink of Chen et al. *(8)*, and the intermediate (sulfidic at depth, SAD) sink of Dahl et al. *(100)* and Kendall et al. *(73)*.

The definition of **euxinic or sulfidic sink** is relatively uniform, referring to settings where dissolved H_2S is persistently present at significant concentrations in the overlying water column *(8)*, typically as a result of hydrographic restriction, as observed in the modern Black

Sea and Cariaco Basin. Some previous studies have included OMZ settings on open ocean upwelling margins in a more broadly-defined euxinic sink, defined by the presence of H_2S in *either* the water column or in shallow pore waters *(104)*. But the authigenic Ni isotope compositions of modern organic-rich upwelling margin sediments *(12, 15, 28)* are very different from those found in the one major modern basin studied to date where H_2S is present in the deep water column (the Black Sea) *(11)*. In upwelling settings, Ni is transported to the sediment via uptake into organic matter in the photic zone, and then transferred to sulfide within the sediment *(12, 15, 26, 28, 106)*. These organic-rich upwelling margin sediments have been shown to faithfully record global seawater δ^{60}Ni, regardless of relative position of the OMZ *(12, 15, 28)*. In contrast, the Ni isotope composition in basins like the Black Sea, with a thin oxic upper water column and a thick H_2S-bearing deep basin, appear to be controlled by a Mn-oxide shuttle that transfers isotopically light Ni across the chemocline, Ni that is then sequestered to sediment *(11)*. Thus, we separate the upwelling and euxinic sinks here. We note, however, that the Mo isotope compositions of these two sinks are much more similar than for Ni *(21)*, this contrast between the two systems for this setting being an example of the potential benefits of considering both isotope systems together in studies of Earth history.

The **oxic sink** is defined as environments in which dissolved oxygen penetrates deeper than 1 cm below the sediments-water interface *(8, 102)*. Such a setting is dominated by authigenic Mn oxide phases. The oxic sink here is identical to the strongly oxic sink defined by Chen et al. *(8)*.

The global areal fractions of each sink reported in previous studies are variable, ranging from 0.35% to ~2% for the upwelling sink, 0.05% to 0.12% for the euxinic sink, and 83% to 96% for the oxic sink *(8, 102, 104, 107-109)*. Although continental margins can underlie up to ~15% of ocean surface area *(107, 109-111)*, these settings are simply defined by depth isobaths and do not correspond to the geochemical classification used here. From a geochemical perspective, OMZ bottom waters with < 0.5 ml O_2 L^{-1} have been proposed to cover ~2% of continental margins and thus ~0.3% of ocean surface area *(111)*. In this study, we propose best-estimate areal fractions of 1.1% for upwelling sinks, 0.08% for euxinic sinks, and 90% for oxic sinks, falling close to the middle of the ranges reported in previous studies. Note that these percentage areas do not add up to 100% because, following *(102)*, a significant portion of the ocean floor does not represent a sink for Mo, and presumably for Ni, due to shallow oxygen penetration depths (<1 cm) and the complete mobilisation of Mn oxide back to the water column. The choice of areal fraction does not affect the modern Ni and Mo budgets: the budgets are determined by the requirement for global elemental and isotope mass balance, so that the assigned areal fraction simply determines an accumulation rate in each sink.

Oceanic Ni mass balance

The Ni isotope composition of the global deep ocean (>500m) is homogeneous at δ^{60}Ni = +1.34 $\pm$ 0.06‰ (mean $\pm$ SD) *(18, 29)*. Though the Ni-depleted low latitude surface ocean is isotopically heavier, this has an insignificant effect on the global ocean Ni isotope composition. Our current knowledge of the modern oceanic mass balance of Ni and its isotopes is summarised in Table 1 and Fig. S1, to which the text refers as appropriate. Note that these

numbers are slightly different from those in Fleischmann et al. *(16)* (see their Fig. 1) due to a small change in the Ni/TOC ratios used to estimate the size of the upwelling and euxinic sinks, as discussed further below.

Inputs – rivers and dust

Cameron and Vance *(30)* reported the dissolved Ni isotope composition of some of the world's major rivers, with the average values for individual rivers varying from +0.36 to +1.27‰, and the discharge- and concentration-weighted average δ^{60}Ni of riverine input being +0.8‰ (Fig. S1 and Table 1). The discharge-weighted total Ni flux was estimated to be 3.6 $\times$ 10^8 mol/yr *(30)*. Thus, the δ^{60}Ni of rivers is isotopically heavier than the upper continental crust (UCC; +0.12 $\pm$ 0.11‰ (1SD)) *(55, 56)* due to isotope partitioning between the dissolved phase and secondary particulate phases formed in the weathering environment and partially retained in soils *(55, 68)*. Dust is a minor source of Ni to the ocean, at 7.6 $\times$ 10^6 mol/yr, and is assumed to have the same Ni isotope compositions as the UCC *(12)*. It should be noted that, based on available evidence, hydrothermal activity is not a significant source of Ni to the modern ocean *(16)*; instead, proximal hydrothermal settings act as Ni sinks, as discussed below.

Overall, Ni sources to the ocean, overwhelmingly dominated by the riverine input, constitute an input to the ocean of 3.68 $\times$ 10^8 mol/yr, with an isotope composition of +0.79‰ (Fig. S1 and Table 1), and are isotopically lighter than that of the ocean. This indicates that isotope fractionation must occur into sinks, if the ocean is to be in steady-state.

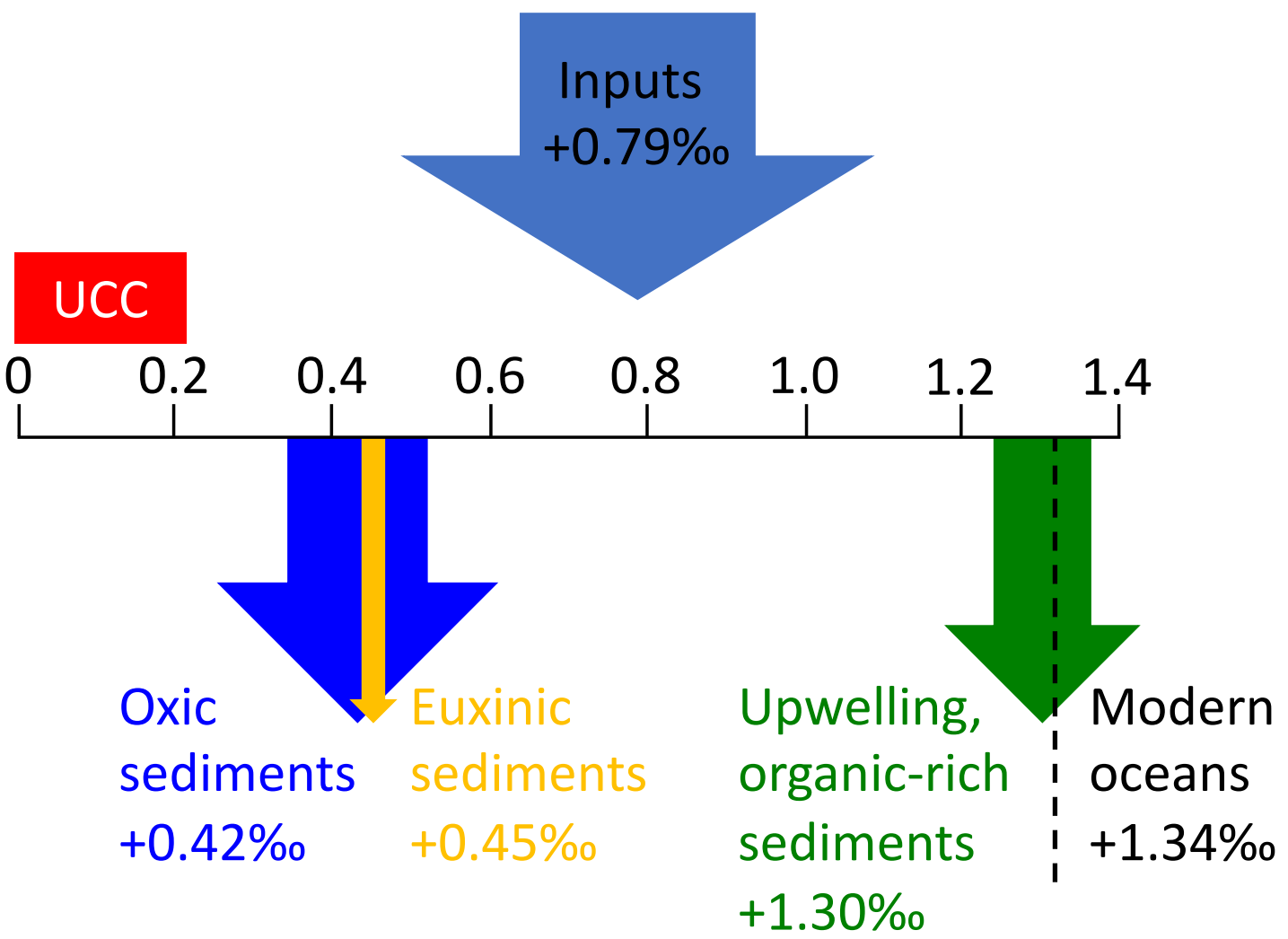

Figure S1: The oceanic Ni isotope budget. The isotope composition (scale shown by black line with tick marks) of the different reservoirs and fluxes, as well as the UCC, are from Cameron and Vance *(30)*; Vance et al. *(11)*; Ciscato et al. *(12)*; Revels et al. *(55)*; Lemaitre et al. *(29)*; He et al. *(15)* and Fleischmann et al. *(16)*. The size of the arrows represents the estimated relative magnitudes of the Ni mass fluxes, and derive from the same studies. The size of the arrows represents the estimated relative magnitudes of the Ni mass fluxes, and derive from the same studies.

Sinks – the upwelling sink

Nickel concentrations are tightly and linearly correlated with TOC in modern organic-rich sediments from upwelling margins (Fig. S2a), and this systematic Ni-TOC relationship suggests that Ni uptake into cells, transfer from the photic zone to the sediment, and burial of organic-associated Ni dominates Ni in upwelling margin sediments *(12, 15, 26, 112)*. In modern upwelling settings, deeper sediments (below ~10 cm) exhibit a relatively constant Ni/TOC ratio at ~9 ppm/wt%, which exceeds the values in phytoplankton, particles in the overlying water column, and surface sediments. The high sedimentary ratio likely reflects the preferential loss of organic carbon relative to Ni, through respiration, with the latter retained in sulfide minerals in the sediment *(12, 26, 28, 112)*. *(113)* estimate that 1.0×10^{13} g C_{org}/yr of organic carbon is buried in these high-productivity zones - a value consistent with that for marine organic carbon buried in settings with <50 μM oxygen along continental margins *(114)*. Though the slope of the correlation in Fig. S2a (for all upwelling sediments, including core-tops) is slightly lower, we take the value of 9 (deeper sediments, after early diagenetic processes) *(112)* as the buried Ni/TOC ratio. With the uncertainty from the regression and the organic carbon burial rate on upwelling margins, the Ni output to this sink can be estimated to be $1.53 \pm 0.06 \times 10^8$ mol/yr (Fig. S1 and Table 1).

Authigenic δ^{60}Ni in organic-rich sediments with high authigenic Ni enrichments (Ni/Al > 8 ppm/wt%) obtained after a detrital correction is +1.30 $\pm$ 0.06‰ for modern upwelling margin sediments (Fig. S1 and Table 1), which is indistinguishable from the Ni isotope composition of modern seawater *(12, 15, 28)*. Therefore, as well as constituting an important sink in the global budget, organic-rich sediments on upwelling margins have the potential to be archives of Ni isotope compositions of contemporary seawater *(12, 15, 28)*.

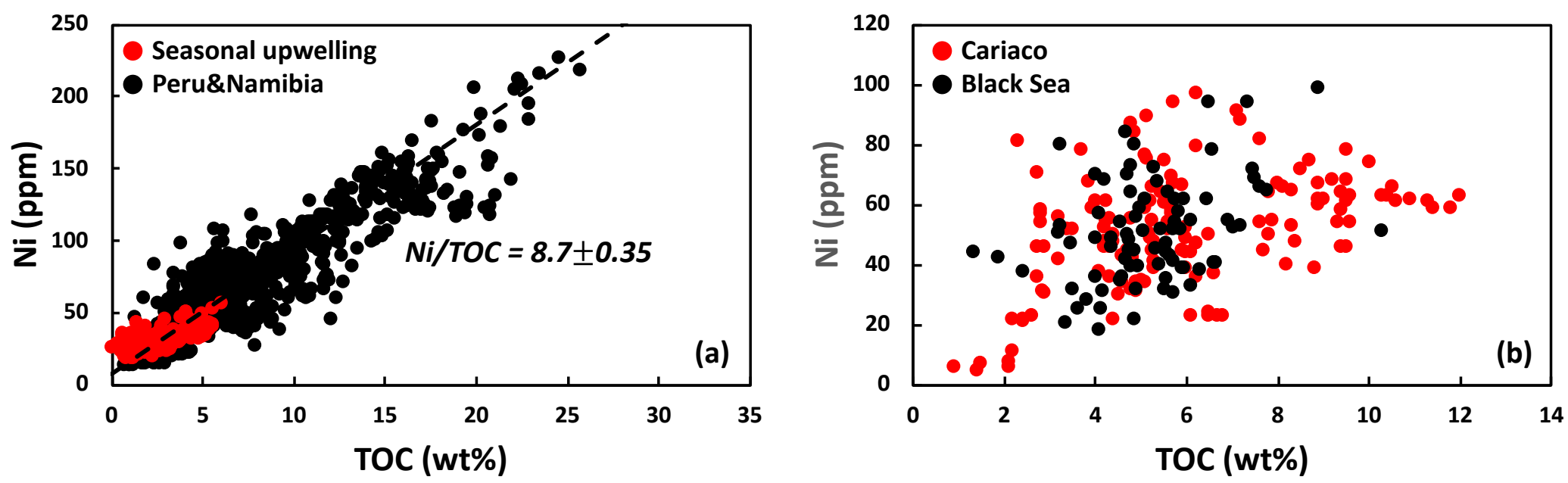

Figure S2: Bulk Ni versus TOC for organic-rich sediments for upwelling (a) and euxinic settings (b). In panel (a), data for the Peru and Namibian upwelling margins (shown in black) are from Böning et al. *(58)*; Borchers et al. *(115)*; McManus et al. *(116)*; Scholz et al. *(117)*, Böning et al. *(26)*; Little et al. *(118)*; Salvatteci et al. *(119)*; Ciscato et al. *(12)*; Abshire et al. *(120)*; Plass et al. *(121)*, Kossack et al. *(122)*; He et al. *(15)*. Data for the Chilean upwelling margin, Gulf of California, Arabian Sea (shown in red) are from Brumsack *(123)*; Böning et al. *(124, 125)*; McManus et al. *(116)*; van der Weidjen et al. *(126)*. In panel (b), data for the Cariaco Basin in red are from Piper and Dean *(127)*; Little et al. *(118)*; Brüske et al. *(128)*. Data for the Black Sea in black are from Luschen et al. *(129)*; Little et al. *(118)*; Brüske et al. *(128)*. The slopes and uncertainties in this study (Figs. S2, S4-S7) are obtained by orthogonal distance regression (ODR) performed using Python. The slopes are reported with 2 standard error (2SE) external uncertainties, calculated by multiplying the internal 2SE by the square root of the mean squared weighted deviation (MSWD) to account for excess scatter indicated by MSWD > 1.

Sinks – the euxinic sink

The Black Sea is currently the only euxinic basin for which both Ni abundance and isotope data are available for the water column and sediments, and sedimentary authigenic δ^{60}Ni is much lighter than seawater, at +0.45 $\pm$ 0.15‰ *(11)* (Fig. S1 and Table 1). These authigenic Ni isotope compositions are attributed to two linked processes, although how they act together requires further investigation: (1) the transfer of Ni to the deep sulfidic basin via the Mn oxide particulate shuttle; (2) partial removal to sediment, perhaps as a sulfide. In the first process, Mn oxides sorb isotopically light Ni in the upper oxic zone and subsequently release it into the deep sulfidic water column *(11, 27)*. Further Ni isotope fractionation may occur between different dissolved species of Ni in the deep sulfidic water column, as lighter Ni isotopes are preferentially partitioned into aqueous Ni sulfide species *(11, 130)*. Given these multiple processes, it is not surprising that the relationship between Ni and TOC for euxinic sediments from both the Black Sea and the Cariaco Basin is much weaker than for upwelling settings (Fig. S2b). However, the vast majority of the samples have Ni/TOC ratios between 5 and 15 ppm/wt%, consistent with the fact that a regression of the data in Fig. S2b, forced through the origin, yields a Ni/TOC ratio of 9.7 $\pm$ 1.2 ppm/wt%. Given an estimated rate of organic carbon burial in euxinic settings globally of 1.0 $\times$ 10^{12} g C_{org}/yr *(113)*, the Ni output to euxinic basins can be estimated to be about 1.7 $\pm$ 0.85 $\times$ 10^{7} mol/yr (Fig. S2 and Table 1). Though this uncertainty is large and further Ni isotope data from other euxinic basins are required, the Ni output to the euxinic basin sink is much smaller than to other Ni sinks, at least in the modern ocean, and has little impact on the Ni mass balance.

Sinks – the oxic sink

The most significant Ni sink from the oceanic dissolved pool is that to open ocean Mn-oxide rich sediments. Fig. S1 shows the required size and isotope composition of this sink, given the information above on inputs and the other two outputs, to achieve isotope and elemental steady-state. This calculation results in a Ni output to this sink of 1.98 $\times$ 10^{8} mol/yr and a Ni isotope composition of +0.42‰ (Fig. S1 and Table 1).

Fleischmann et al. *(16)* demonstrate that a partitioning of the global Mn oxide output into an abyssal pelagic setting with a Ni/Mn around 0.02 g/g and the measured δ^{60}Ni of abyssal pelagic sediments (+0.68 ± 0.24‰), and a proximal hydrothermal setting with Ni/Mn ratios of 0.0004-0.002 (within the measured range) and the measured δ^{60}Ni of hydrothermal sediments (-1.06 ± 0.24‰) rationalises the blue arrow in Fig. S1. In this view, because of the very different Ni/Mn ratios in the two different Mn-oxide sinks, 50-90% of Mn is buried in proximal hydrothermal sediments, whereas 85-90% of Ni burial occurs via the abyssal pelagic sink.

Oceanic Mo mass balance

Molybdenum (Mo), with its isotope system (δ^{98}Mo), is among the most extensively studied trace metals in both modern and ancient oceans. Molybdenum enrichments in modern sediments result from multiple mechanisms. In upwelling sinks, Mo accumulation is primarily driven by diffusion down a concentration gradient from the water column to a sink at depth where dissolved H_2S sequesters Mo to the sediment via the conversion of soluble molybdate (MoO_4^{2-}) to insoluble Mo thiomolybdate ($MoO_xS_{4-x}^{2-}$) *(131)*. Additional enrichment in specific situations is attributed to Mn or Fe oxide particulate sources *(103, 122, 132)*. In restricted

basins with sulfidic water columns, this latter process occurs in the water column, with the extent of enrichment largely depending on the degree of restriction and Mo supply from the open ocean *(132)*. In the fully oxic open ocean, Mo is removed by sorption onto Mn oxides *(107, 133)*.

The global input of Mo has an average isotope composition of +0.70‰ *(72)*, with a total flux of 3.3 × 10^8 mol/yr. This includes a riverine input of 3.1 × 10^8 mol/yr and a low-temperature hydrothermal input of 0.2 × 10^8 mol/yr *(73)*. The Mo isotope fractionations between different sinks and the global oceanic dissolved pool are adopted from Chen et al. *(8)*, with minor modifications to the output flux for each sink (Fig. S3). Recent studies have proposed that the Mo isotope compositions of euxinic sinks and upwelling sinks are indistinguishable, particularly for intermidiate euxinic sinks *(21)*. Here, however, the euxinic sinks are defined – consistent with the definition adopted above – by persistent high dissolved H_2S concentrations in the overlying water column. Under such conditions, the Mo isotope compositions of euxinic sinks are isotopically heavier than those of upwelling sinks, consistent with observations from the modern Black Sea and Cariaco Basin *(128)*.

In this study, as in those of Chen et al. *(8)* and Kendall et al. *(73)*, the Mo output to oxic sinks is higher than reported by Scott et al. *(104)* and Reinhard et al. *(102)*, who based their estimates on a constant Mn accumulation rate in fully oxic Mn-rich abyssal sediments *(25, 134)* and the relatively constant Mo/Mn ratios in Mn oxide-rich sediments *(135)*. However, Mn accumulation rates in the open ocean are highly variable and can be up to two orders of magnitude greater in proximal hydrothermal sediments compared to the abyssal ocean *(25)*. As a result, the mass balance schemes of Scott et al. *(104)* and Reinhard et al. *(102)* bury much too little isotopically light Mo in the Mn oxide sink in order to balance the isotope budget.

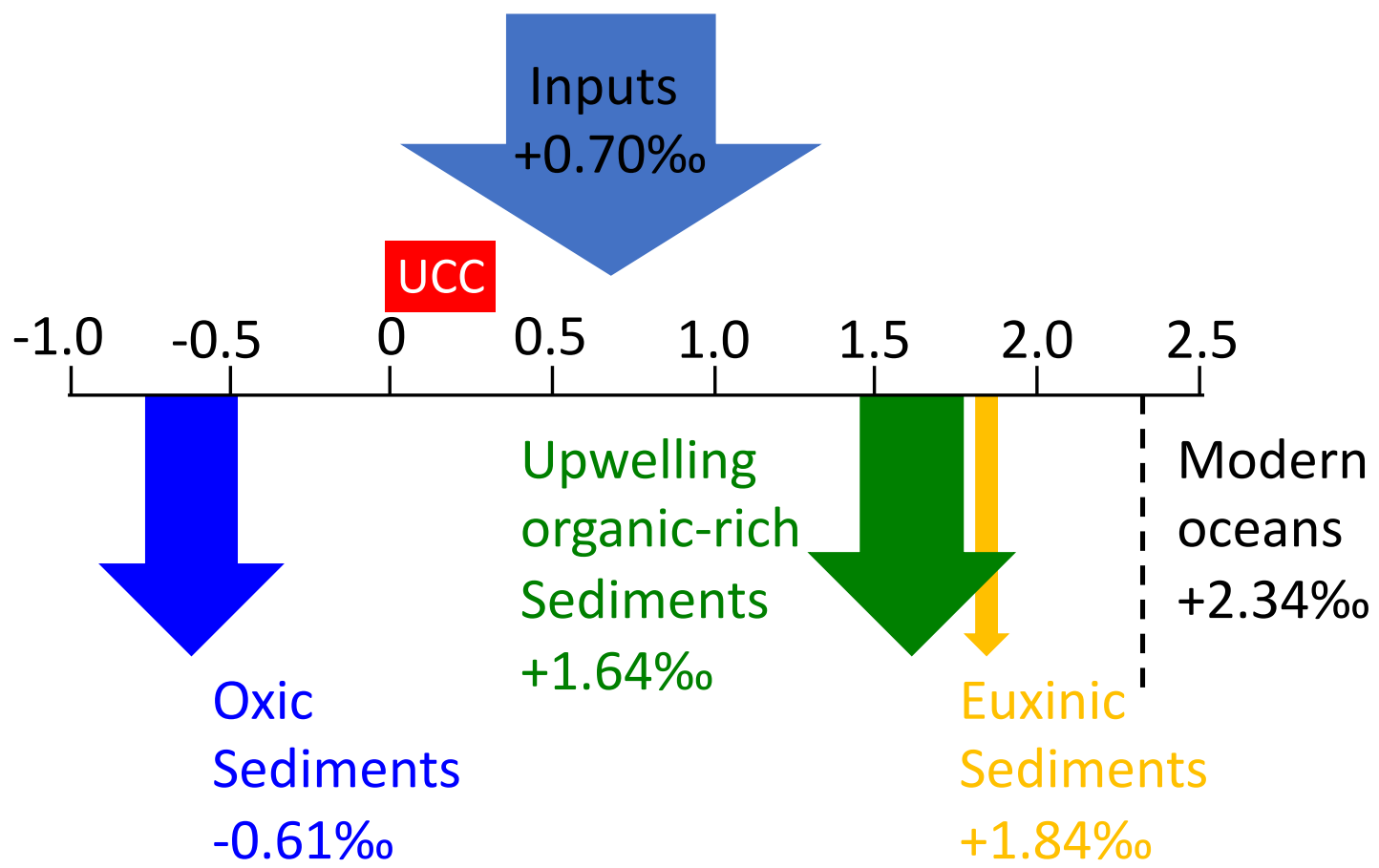

Figure S3: The oceanic Mo isotope budget. The isotope composition (scale shown by black line with tick marks) of the different reservoirs and fluxes, as well as the UCC, are from Siebert et al. *(136)*; Archer and Vance *(72)*; Chen et al. *(8)*; Kendall et al. *(73)*. The size of the arrows represents the estimated relative magnitudes of the Mo mass fluxes, and derive from the same studies.

The Mo output flux to euxinic restricted basin sinks is considerably smaller and more tightly constrained than that to oxic and upwelling sinks, but can appear larger in studies that include margin sediments underlying O_2 poor waters and with sulfidic porewaters. Given the relatively small size of euxinic sinks, the Mo isotope fractionation into euxinic settings has

much less influence on the modern oceanic Mo mass balance *(8, 100)*. In this study, a Mo output flux of 2.0×10^7 mol/yr to euxinic sinks is used, consistent with that reported in previous studies *(73, 102, 105)*. The Mo outputs to the upwelling and oxic sinks are calculated by maintaining steady-state elemental and isotopic mass balances in the modern ocean (Fig. S3 and Table 1).

Observed Ni/Mo ratios in modern ocean sediments

Mutually-consistent Ni and Mo budgets in the modern ocean ought to be compatible with measured Ni/Mo ratios in the sediments that represent the three sinks. Here, we compile sedimentary Ni and Mo concentration data across various redox settings in the modern ocean to assess this. By combining Ni and Mo data with the most comprehensive understanding of organic carbon and Mn output fluxes, we calculate characteristic authigenic Ni/Mo ratios using multiple approaches. We then compare these Ni/Mo ratios with the corresponding output flux ratio for each redox setting derived from our oceanic Ni and Mo budgets.

We apply two approaches to estimate the authigenic Ni/Mo ratios in non-oxic (upwelling and euxinic) sinks. In the first approach, Ni and Mo concentrations are separately plotted against total organic carbon. The authigenic Ni/Mo ratio is calculated as the ratio of the slopes of Ni/TOC and Mo/TOC obtained from the resulting regression. In the second approach, authigenic Ni is plotted against authigenic Mo after correcting for detrital inputs, and the authigenic Ni/Mo ratio is derived from the slope of the resulting regression. These two approaches allow for a cross-validation of authigenic Ni/Mo ratio estimates in non-oxic settings.

Observed Ni/Mo ratios in upwelling sinks

For the modern upwelling sink, in addition to the perennial upwelling margins such as Peru and Namibia, more seasonal upwelling margins - including the Chilean and California Margins and Arabian Sea - are also considered (Figs. S2a, S4a, S5). Notably, data from seasonal upwelling margins fall on the main Ni-TOC, Mo-TOC, and authigenic Ni-Mo trends, but are consistently located at the lower ends (Figs. S2a, S4a, S5). As described earlier, a Ni/TOC ratio of 9.0 ± 0.35 is used for upwelling sinks.

The Mo-TOC relationship for upwelling sediments (Fig. S4a) is more complex than that observed for Ni and TOC. Unlike for Ni, there is no direct, mechanistic, link between the accumulation of Mo and organic carbon in upwelling sediments. Rather, upwelling regions with high primary productivity export the organic carbon to sediment that leads to porewater sulfate reduction and the sulfide that fixes Mo into the solid sediment. We investigate Mo-TOC correlations here simply as a potential tool for obtaining Mo/TOC ratios in these sediments, to couple with Ni/TOC to obtain Ni/Mo ratios. On Fig. S4a, three primary trends can be identified, including two roughly parallel trends with similarly low Mo/TOC slope, as indicated by the black dashed lines. The lower black dashed trend passes through the origin, while the upper black dashed marks samples with systematically higher Mo concentrations for a given TOC (Fig. S4a).

The steeper trend on Fig. S4a, with a slope corresponding to a Mo/TOC ratio of 15.1 ± 1.2 ppm/wt%, explains over 80% of the data (n>1100). The data lying to the right of this main trend derive almost entirely from the 120-350m interval on the Peru Margin, a depth interval over which mass accumulation rates are at a minimum and where the burial efficiencies of particulate organic carbon are higher than on the shelf. On the other hand, the main steep trend might suggest an additional enrichment mechanism for Mo. This enrichment may reflect the contribution of a Mn-oxide particulate shuttle to Mo accumulation *(122, 137)* and/or lateral transport and redeposition *(120)*.

Though the precise interpretation of the complexity in the Mo-TOC data remains speculative, over 80% of the data align closely with the steep Mo/TOC trend. We therefore interpret the Mo/TOC ratio of 15.1 ± 1.2 ppm/wt% from the black solid line in Fig. S4a as the value resulting from all potential enrichment mechanisms for Mo in modern upwelling margins. When combined with the constant Ni/TOC ratio of 9.0 ± 0.35 ppm/wt%, this yields an authigenic Ni/Mo ratio of 0.60 ± 0.05 g/g, or 0.97 ± 0.09 mol/mol, in upwelling sinks, closely matching the molar ratio of 0.90 required to mutually balance Mo-Ni budgets.

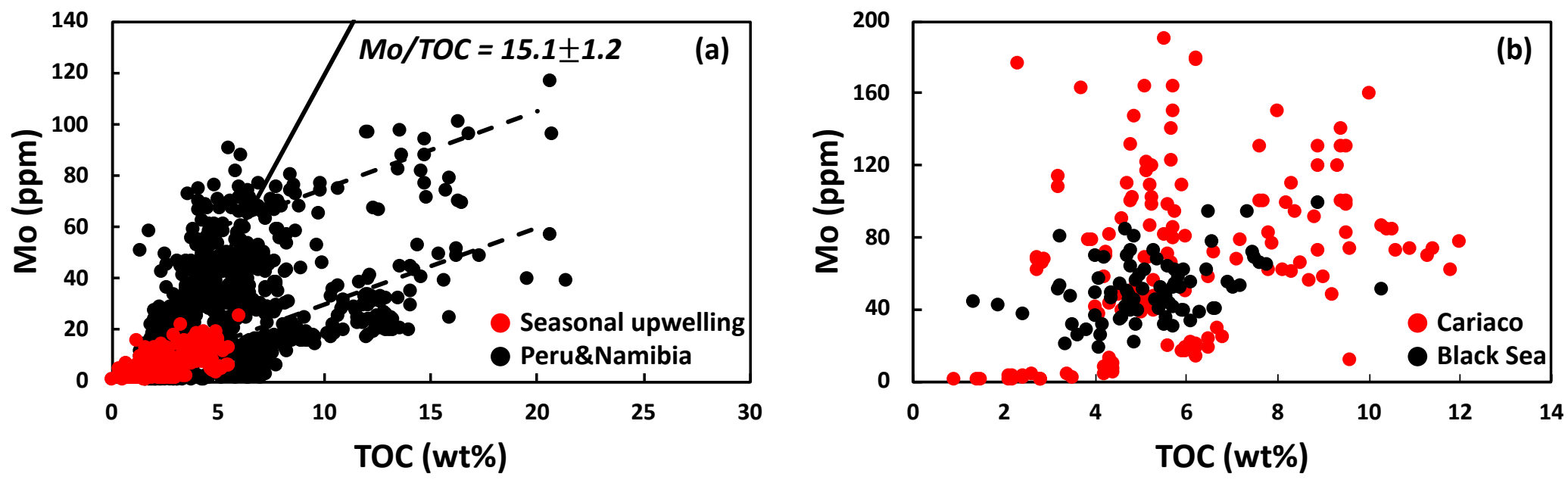

Figure S4: Bulk Mo versus TOC for organic-rich sediments for upwelling (a) and euxinic settings (b). In panel (a), data from the Peru and Namibian upwelling margins is shown in black, and data from the Chilean upwelling margin, Gulf of California, Arabian Sea are shown in red. In panel (b), data from the Cariaco Basin are shown in red, and data from the Black Sea are shown in black. See caption to Fig. S2 for data sources.

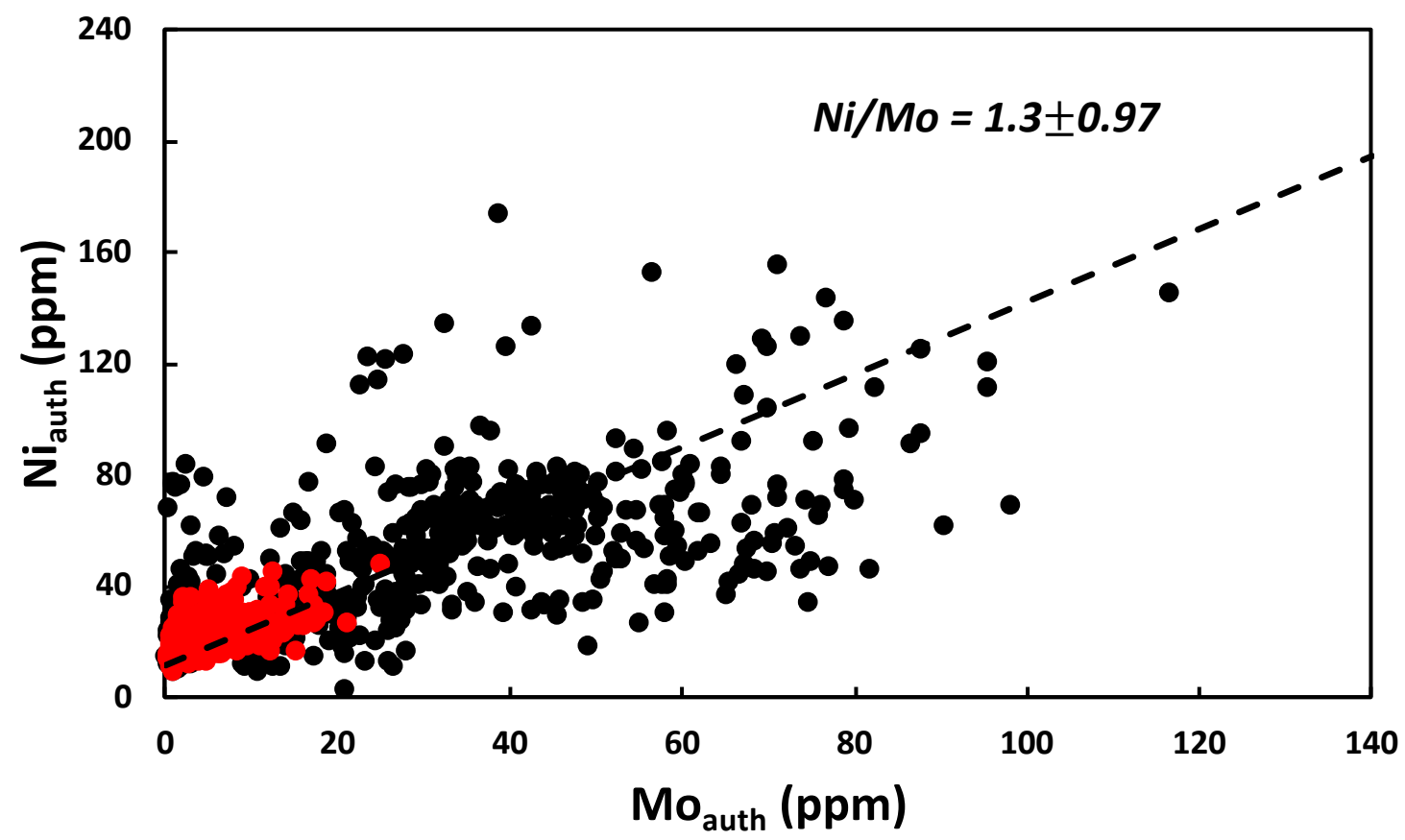

Figure S5: Plot of authigenic Ni versus authigenic Mo concentrations for organic-rich sediments from upwelling settings. Authigenic concentrations (corrected for detrital contribution) calculated as $X_{auth} = X_{sample} - [Al_{sample} \times (X/Al)_{detrital}]$, where Ni/Al = 1.7×10^{-4} g/g *(26)* Mo/Al= 0.14×10^{-4} g/g *(138)*. Data for the Peru and Namibian upwelling margins are shown in black, while data for the Chilean upwelling margin, Gulf of California, and Arabian Sea are shown in red. See caption to Fig. S2 for data sources.

This same issue may impact the other method we explore here. The authigenic Ni/Mo ratio, derived from the regression of authigenic Ni and Mo (Fig. S5), is 1.3 $\pm$ 0.97 g/g, corresponding to a molar Ni/Mo ratio of 2.7 $\pm$ 1.6. This value is much higher than that required by the global mass balances, of 0.9 mol/mol. However, there is significant scatter in the relationship in Fig. S5, leading to a large uncertainty in the slope and a Ni/Mo ratio. Again, the Mn particulate shuttle and or lateral transport may variably enhance Mo accumulation at a given TOC - and thus at a given Ni level, and consequently, the true Ni/Mo ratio is highly likely lower than that derived from the regression of all authigenic Ni and Mo concentrations shown in Fig. S5.

Observed Ni/Mo ratios in euxinic sinks

In euxinic sinks, our proposed modern ocean Ni and Mo budgets require a molar Ni/Mo ratio of 0.85. In addition to the well-studied modern Black Sea, a strongly restricted euxinic setting, the modern Cariaco Basin, a semi-restricted euxinic setting, is also included to evaluate observed Ni/Mo ratios in modern euxinic sinks.

As noted earlier, the regression-derived Ni/TOC ratio in euxinic sinks is 9.7 $\pm$ 1.2, although the Ni-TOC relationship exhibits significant scatter. The Ni-TOC data show no substantial difference between the modern Black Sea and the Cariaco Basin (Fig. S2b). Molybdenum concentrations for the Cariaco Basin are clearly displaced to higher values at any given TOC than those for the Black Sea (Fig. S4b). This difference can probably be attributed to the lower Mo inventory in the Black Sea due to its strong restriction *(132)*, and to the operation of a particulate shuttle in the Cariaco basin. The Mo-TOC plot also exhibits significant scatter due to these complexities. Taking the same approach as with Ni-TOC for euxinic basins, a regression of the data in Fig. S4b, forced through the origin, yields an estimate of the Mo/TOC ratio of 14.2 $\pm$ 4.2. Together with the Ni/TOC ratio, this would correspond to a Ni/Mo ratio of 0.68 $\pm$ 0.22 g/g or 1.1 $\pm$ 0.36 mol/mol.

Regression of authigenic Ni versus Mo in euxinic sediments reveals a trend that does not go through the origin (Fig. S6) and yields an authigenic Ni/Mo ratio of 0.23 $\pm$ 0.49 g/g, or 0.38 $\pm$ 0.80 mol/mol. The positive y-axis intercept is particularly forced by the Cariaco data, and may reflect the complex enrichment mechanisms that are likely reflected there: upwelling and high productivity (enriching Ni but not Mo), a Mn oxide particulate shuttle (higher Ni/Mn ratio than Mo/Mn in Mn oxides; see the following section), sulfidation of metals in the deep basin. The Black Sea data on their own define a correlation with an intercept closer to the origin (y-intercept = 18), and with a slope that suggests a molar Ni/Mo ratio of 1.1.

Overall, though uncertainties are again large due to complexities for both metals in the euxinic sink, these estimates of Ni/Mo ratios of 0.4-1.1 mol/mol bracket the value required by the coupled mass balance of 0.85. Importantly, as noted earlier, the output fluxes of Ni and Mo to euxinic sinks are both small in the modern ocean and have little influence on the oceanic mass balance.

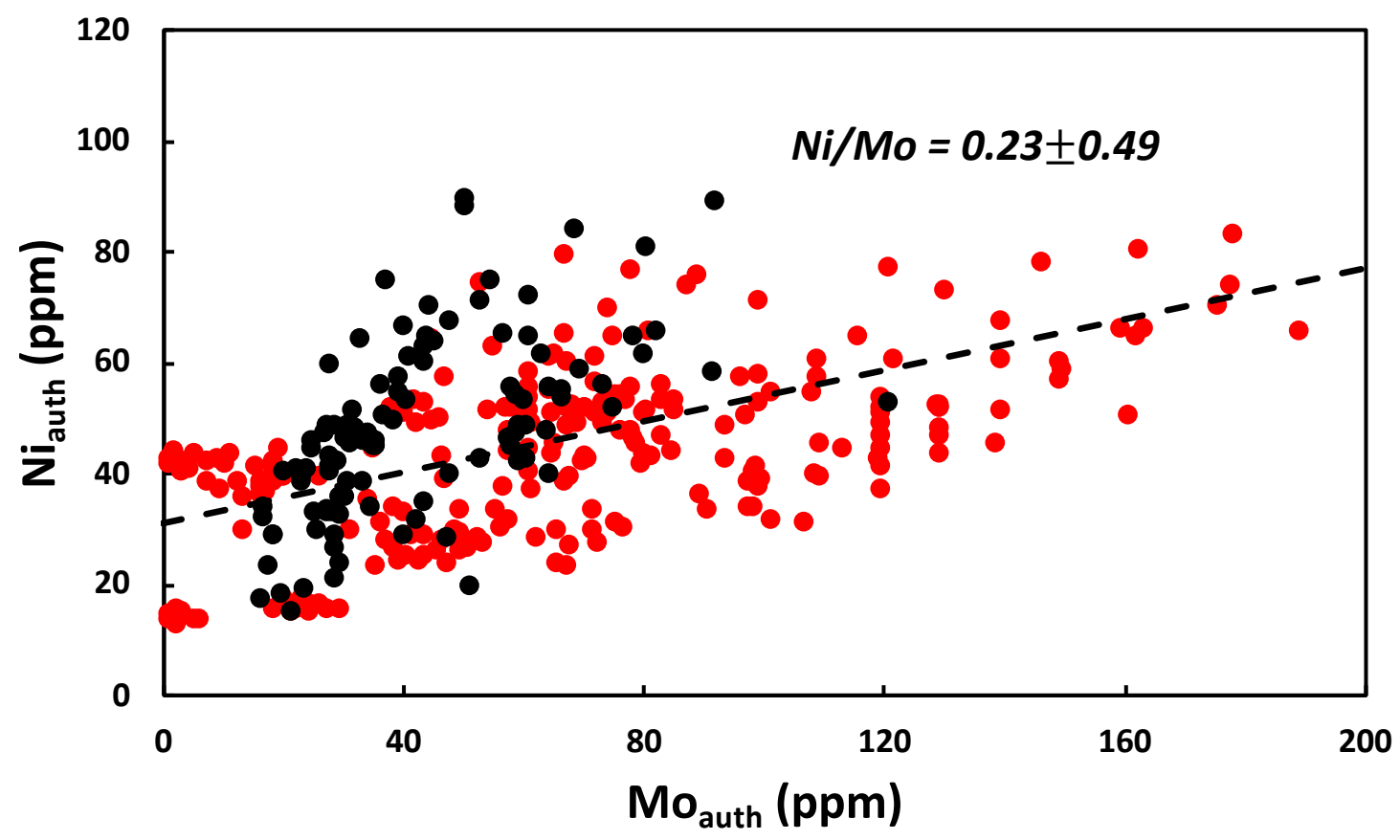

Figure S6: Plot of authigenic Ni versus authigenic Mo concentrations for organic-rich sediments for euxinic settings. Data from the Cariaco Basin are shown in red, and data from the Black Sea are shown in black. See caption to Fig. S2 for data sources.

Observed Ni/Mo ratios in oxic sinks

To estimate the authigenic Ni/Mo ratio for the oxic sink, we follow an analogous approach to that used for non-oxic sinks, where Ni and Mo concentrations are correlated with organic carbon. In oxic settings, including the proximal hydrothermal and abyssal pelagic sub-sinks *(16)*, however, authigenic Mn oxides are the dominant host phase for both Ni and Mo. Therefore, our estimation of the Ni/Mo ratio in oxic sinks is based on Ni/Mn and Mo/Mn ratios. It has been suggested that Mo has a marked affinity for Mn, and that the Mo/Mn ratio in Mn oxide-rich sediments remains relatively constant at 0.002 g/g. This includes Mn crusts, nodules and Mn-rich pelagic sediments *(135)*. This relationship is further supported by a broader compilation of Mo and Mn data from oxic sediments, as shown in Fig. S7. All oxic sediments lie close to the main Mo-Mn slope of 22 ± 5.4 ppm/wt%, though with more scatter observed for proximal hydrothermal sediments. We therefore assume that Mo accumulation rates in oxic settings scale linearly with global Mn accumulation rates, and apply a Mo/Mn ratio of 0.002 g/g to both proximal hydrothermal settings and abyssal pelagic settings.

In contrast, the behavior of Ni relative to Mn is more complex *(16)*. Although Ni also has a strong affinity for particulate Mn oxides, Ni/Mn ratios in Mn oxide-rich sediments show a bimodal distribution, with notably higher values observed in abyssal pelagic settings than in proximal hydrothermal settings. Fleischmann et al. *(16)* attributed this difference to faster accumulation rates in proximal hydrothermal settings, such that Ni sequestration to Mn oxides in such settings is dominated by sorption during rapid Mn oxide precipitation and rapid burial. In contrast, when precipitation and accumulation rates are slower Ni can be also gradually incorporated into the structure of Mn phases, leading to the further enrichment of Ni in solid phases beyond initial surface adsorption *(16, 139)*.

A further challenge arises from the high variability of Mn accumulation rates in the open ocean, making estimates of the global average Mn accumulation rate contentious *(25, 104, 140, 141)*. In particular, Mn accumulation rates in the proximal hydrothermal setting can be more than 60 times greater than that in the abyssal pelagic setting *(25)*. To account for these variabilities, we separately calculate the Ni/Mn and Mo/Mn ratios for proximal hydrothermal

settings and abyssal pelagic settings, and then derive a global flux-weighted average Ni/Mo ratio for the oxic sink.

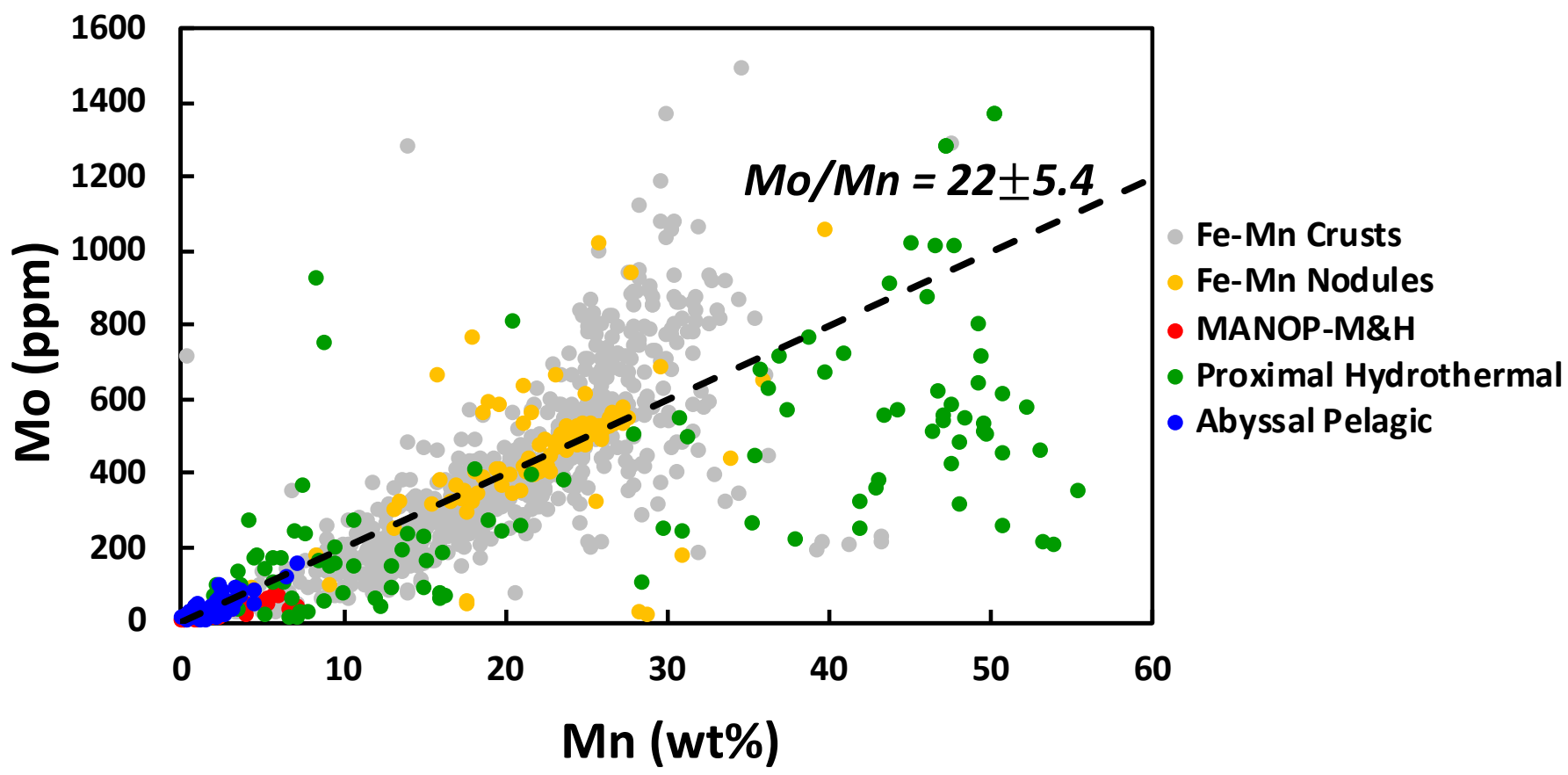

Figure S7: Bulk Mo versus Mn for oxic sediments. Data for the Fe-Mn crusts in grey are from Manheim and Lane-Bostwick *(142)* and Gueguen et al. *(31)*. Data for Fe-Mn nodules in orange are from Krishnaswami et al. *(143)*; Calvert and Price *(144)*; Calvert and Piper *(145)*; Heller et al. *(146)*; Gueguen et al. *(31)*; Fleischmann et al. *(16)*. Data for abyssal pelagic sediments in blue are from Calvert and Price *(144)*; Shimmield and Price *(135)*; Fleischmann et al. *(16)*. Data for MANOP sites M and H sediments in red are from McManus et al. *(116)*; Poulson Brucker et al. *(105)*; Fleischmann et al. *(16)*. Data for proximal hydrothermal sediments in green are from Boström and Peterson *(147)*; Moorby and Cronan *(148)*; Hein et al. *(149)*; Zeng et al. *(150)*; Pan et al. *(151)*; Goto et al. *(152)*; Gueguen et al. *(31)*; Sun et al. *(153)*.

The Ni/Mn ratio in abyssal pelagic settings is relatively constant at 0.02 g/g *(16)*, while the Mo/Mn ratio in oxic settings is 0.002 g/g (Fig. S7) *(135)*. This yields a corresponding Ni/Mo ratio of 10 g/g, or 16 mol/mol. In contrast, the Ni/Mn ratio in proximal hydrothermal settings is highly variable, ranging from 0.0001 to 0.008 g/g *(16)*. Nevertheless, Fleischmann et al. *(16)* proposed that a Ni/Mn ratio of about 0.0004 g/g can reproduce the required size and isotope composition of the global oxic sink for Ni under the steady-state elemental and isotopic mass balance constraints. Although this low ratio of 0.0004 g/g is not uniquely constrained, it aligns well with Ni/Mn ratios measured in particles near the East Pacific Rise *(154)*.

Applying the best-estimate value of 0.0004 g/g would result in Ni/Mo ratios of 0.2 g/g, or 0.33 mol/mol, for proximal hydrothermal settings. This is much lower than the value for abyssal pelagic settings but closer to the modern seawater Ni/Mo molar ratio of 0.08. This appears consistent with the fact that near mid-ocean ridges, where Mn accumulation rates are extremely high, the ratio of Ni and Mo scavenged by the newly formed Mn oxides is likely controlled by their ambient concentrations in the water column, consistent with first-order rate laws. However, further studies are needed to quantitatively constrain Ni and Mo behavior under such conditions.

Fleischmann et al. *(16)* have suggested that proximal hydrothermal settings, with the Ni/Mn ratio of 0.0004, contribute about 80% of global Mn burial and consequently 80% of Mo burial, given the relatively constant Mo/Mn ratio observed in oxic sinks. However, because of the low Ni/Mn ratio, only about 15% of Ni is buried close to the mid-ocean ridge. Abyssal pelagic settings, where the Ni/Mn ratio is significantly higher at ~0.02 g/g, contribute ~85% of Ni burial and around 20% of Mn and Mo burial in oxic sinks. Using the Ni/Mo ratios of 16 mol/mol

for abyssal pelagic settings and 0.33 mol/mol for proximal hydrothermal settings, respectively, combined with these Ni and Mo burial flux proportions, we estimate the global flux-weighted average Ni/Mo in oxic settings.

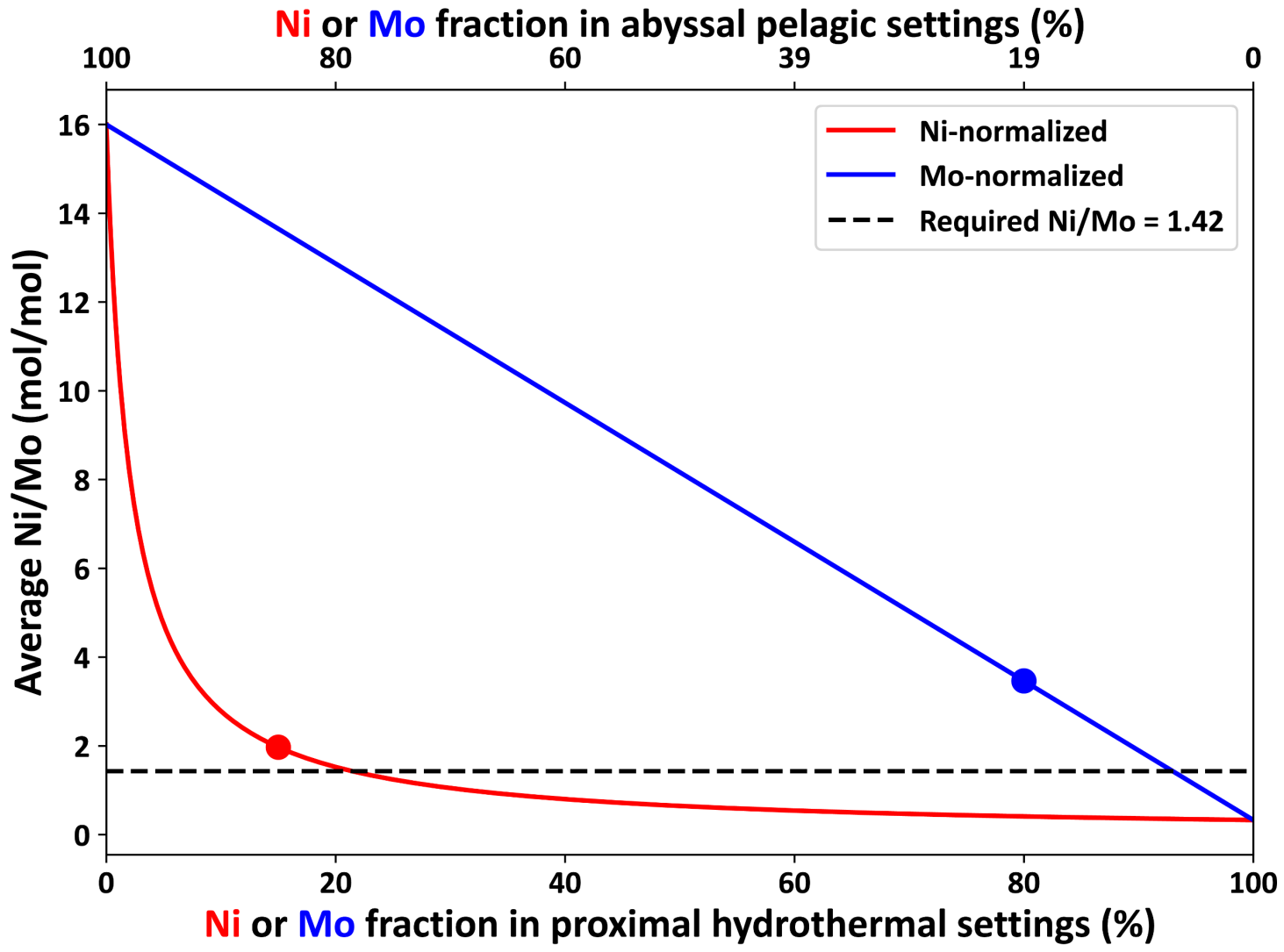

Figure S8: The global flux-weighted average Ni/Mo molar ratio as a function of the mixing fraction between proximal hydrothermal settings and abyssal pelagic settings. The red curve indicates the Ni-normalized mixing scenario, while the blue one is the Mo-normalized mixing scenario. The black dashed line is the required Ni/Mo ratio of 1.42 mol/mol. The red dot shows the case where 15% of Ni is buried in proximal hydrothermal settings in the Ni-normalized scenario, and blue dot shows the case where 80% of Mo is buried in proximal hydrothermal settings in the Mo-normalized scenario.

If the output flux and spatial distribution of Ni in oxic settings are assumed to be better constrained (Ni-normalized scenario, shown by the red curve in Figure S8), the resulting global flux-weighted average Ni/Mo is 1.97 mol/mol (see the red dot in Fig. S8), but this value is highly sensitive to the proportion of Ni buried in proximal hydrothermal settings at the lower end (Fig. S8). For example, increasing the proportion of Ni buried in proximal hydrothermal settings from 15% to 20% would lower the average Ni/Mo ratio to 1.52 mol/mol. Alternatively, if the Mo output flux and distribution are considered better constrained, the global flux-weighted average Ni/Mo in oxic settings is 3.46 mol/mol (see the blue dot in Fig. S8), but the global average Ni/Mo ratio in oxic settings would drop to 1.90 mol/mol as the fraction of Mo buried in abyssal pelagic settings declines from 20% to 10% (shown by the blue line in Figure S8).

Overall, the global flux-weighted average Ni/Mo in oxic settings, calculated independently from the output fluxes and spatial distributions of Ni and Mo, is consistent with ~15% of total Ni being buried in proximal hydrothermal settings, with the remaining ~85% buried in abyssal pelagic settings, and with ~90% of Mo and Mn buried in proximal hydrothermal settings. This would imply a global average Ni/Mo ratio of 1.90 mol/mol, which is close to the required molar Ni/Mo ratio of 1.42 (Table 1). This partitioning of Ni burial between the two oxic sinks is also within the modelled range for proximal hydrothermal and abyssal pelagic settings given uncertainties in Ni/Mn ratios *(16)*.

However, there is also a further uncertainty. The global average Mn accumulation rate of 1.3 μg cm^{-2} yr^{-1} is used in Fleischmann et al. *(16)* to reproduce the size and isotope composition of the oxic sink for Ni that is required by the steady-state elemental and isotopic mass balances. This value was chosen to be consistent with the maximum Mn concentration of high-T hydrothermal fluids and the high-T hydrothermal water flux. However, balancing the modern ocean Mo budget, combining the Mo output flux to oxic settings (1.40×10^8 mol/yr), the constant Mo/Mn ratio of 0.002 g/g and 90% of the modern seafloor covered by oxic sinks yields a corresponding global average Mn accumulation rate of around 2 μg cm^{-2} yr^{-1}. This estimate is higher than the value used by Fleischmann et al. *(16)* but very close to that proposed by van Hulten et al. *(141)*. Note, however, that a higher global average Mn accumulation rate of 2 μg cm^{-2} yr^{-1} will not alter conclusions drawn by Fleischmann et al. *(16)*; it only requires a minor adjustment to the Ni/Mn ratio in proximal hydrothermal sediments from 0.0004 to about 0.0003.

Monte Carlo simulation

It is clear from the above discussion that there are multiple ways of constraining mutually-consistent oceanic budgets for Ni, Mo and their isotopes. These multiple approaches are associated with uncertainties in data, and do not yield perfectly consistent results. In this section we use a Monte Carlo simulation to understand the impacts of these uncertainties and to assess which set of parameters yield the most consistent framework. We performed a Monte Carlo simulation comprising 100 independent runs with different random seeds, each with 10,000 iterations in Python to solve the mutually-consistent modern Ni and Mo budgets given the following steady-state elemental and isotopic mass balance equations:

$$F_{input} = F_{upwelling} + F_{euxinic} + F_{oxic} \quad (1)$$

$$F_{input}\delta_{input} = F_{upwelling}\delta_{upwelling} + F_{euxinic}\delta_{euxinic} + F_{oxic}\delta_{input} \quad (2)$$

where F and δ are the Ni and Mo fluxes and the isotope compositions of the inputs and outputs, respectively.

All Monte Carlo model input values and their standard deviations are provided in Table 1. A relative standard deviation of 10% for fluxes and 0.1‰ for isotope compositions was assigned to all Ni and Mo to account for uncertainties, except where specifically constrained by observations described above. Given that both observed and assigned uncertainties are expressed as standard deviations, all simulations were performed with randomly sampled parameters following normal distributions defined by observed means and standard deviations.

For each iteration, the model draws the fluxes and isotope compositions of Ni and Mo inputs from normal distributions defined by observed means and assigned standard deviations. Mo output fluxes into upwelling, euxinic and oxic sinks are also sampled, with their isotope compositions drawn independently. Nickel output fluxes and isotope compositions into upwelling and euxinic sinks are sampled first, and the Ni flux to oxic sinks and its isotope composition are consequently calculated based on the steady-state mass balance. Furthermore, in each iteration, the model calculates the Ni/Mo molar ratios in the three sinks

and compares these ratios to observationally derived target values for the upwelling and euxinic sinks. The model returns only solutions that satisfy Mo isotope mass balance and yield Ni/Mo flux ratios in upwelling sinks and euxinic sinks within the range observed.

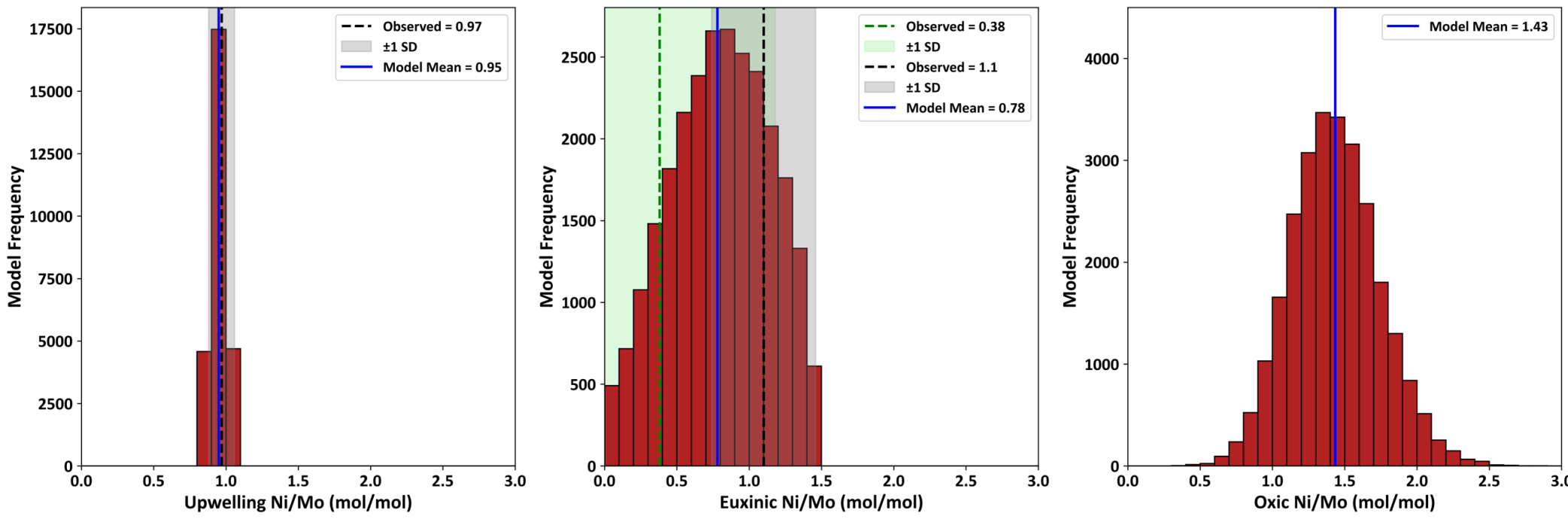

Figure S9: Frequency distributions of Ni/Mo molar flux ratios from Monte Carlo simulations for upwelling, euxinic and oxic sinks. The observed mean Ni/Mo ratio (black and green dashed lines) and their $\pm$1SD uncertainty ranges (gray and green shaded areas) are shown for upwelling and euxinic sinks. The blue solid lines indicate the mean Ni/Mo flux ratio derived from valid model solutions for each sink.

Histograms of Ni/Mo molar flux ratios across all valid iterations are plotted for upwelling, euxinic and oxic sinks in Fig. S9. The frequency distributions of Ni/Mo molar ratios, derived from Monte Carlo simulations for the three sinks, generally exhibit a normal-like distributions. For both upwelling and euxinic sinks, the observed mean Ni/Mo ratios (black and green dashed lines) are very close to the mean values from the valid model solutions (blue lines). Importantly, the mean $\pm$ 1SD and median values for each output parameter across all valid solutions were found to be in excellent agreement with observed values (Table 1). Therefore, in this study, the modelled mean fluxes and isotope compositions for Ni and Mo were applied as their respective marine budgets.

Ni and Mo accumulation rates in modern ocean sediments

Nickel and Mo accumulation rates exhibit over an order of magnitude variability across each individual sink in the modern ocean, largely due to differences in the estimation methods used in previous studies and the inherent heterogeneity of each sink. In upwelling sinks, estimated Ni and Mo accumulation rates range from tens to hundreds of μmol/m^2/yr *(8, 102, 104, 121, 122, 155)*. Euxinic sinks show similar variability, with both Ni and Mo accumulation rates typically within the same range, albeit typically higher than in upwelling sinks *(8, 102, 104, 123, 127, 155-157)*. In oxic sinks, Mo accumulation rates are generally below one μmol/m^2/yr *(102, 104, 155, 158, 159)*, while Ni accumulation rates span a range from a few tenths to tens of μmol/m^2/yr *(25, 143, 155, 160, 161)*. Lower rates are typical of abyssal pelagic settings, with higher rates in proximal hydrothermal settings.

In this study, and specifically for Fig. 3 in the main paper, Ni and Mo accumulation rates in each sink were calculated by dividing the output flux by the area of each sink, and both observed and modelled accumulation rates fall well within the published ranges for each sink (Table 1). These modelled, mutually-consistent modern Ni and Mo budgets, along with the resulting mean accumulation rates, are used to semi-quantitatively constrain changes in the output fluxes in the Late Cretaceous Ocean in the main text.

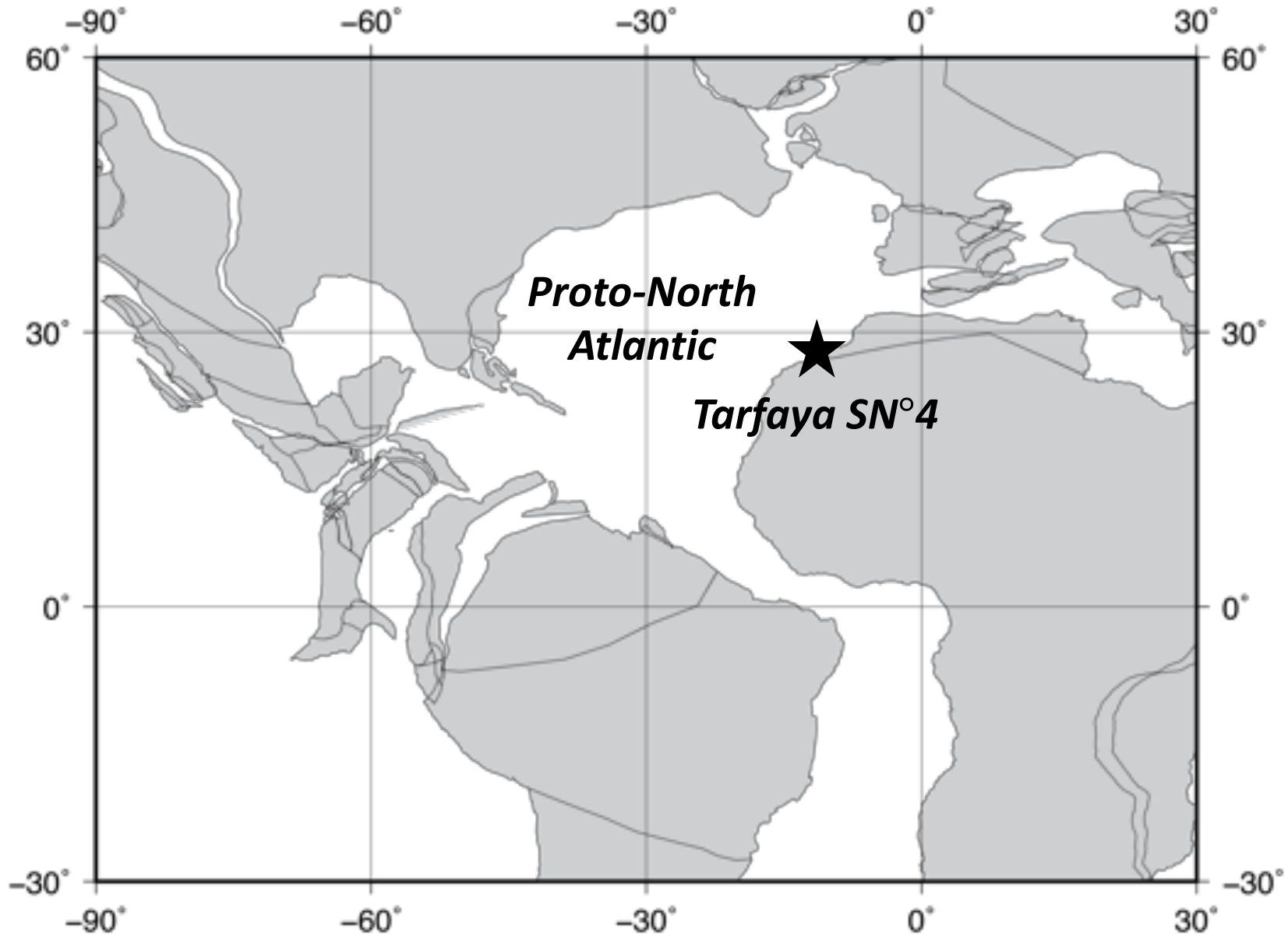

Fig. S10: The location of the Tarfaya Basin core SN°4 (black star) in the proto-North Atlantic, shown on a reconstruction of the Late Cretaceous paleogeography at 94 Ma (ODSN plate tectonic reconstruction service, www.odsn.de).

Table S1. Elemental abundances and Ni isotope compositions of the Tarfaya Basin core SN°4.

Depth	Stage	Al	Ni	δ^{60}Ni	2 σ
m		wt%	ppm	‰	‰
42.12	Turonian	1.0	80.7	1.01	0.04
45.11	Turonian	1.4	79.5	0.95	0.05
46.29	Turonian	1.6	74.4	0.88	0.03
49.83	Turonian	0.8	85.6	1.06	0.04
51.42	Turonian	2.7	99.9	0.91	0.04
54.30	Turonian	2.0	110	0.84	0.05
56.55	Turonian	2.1	130	1.10	0.04
58.93	Turonian	1.8	113	1.14	0.04
61.91	Turonian	2.3	35.4	0.86	0.04
67.52	OAE 2	2.5	83.0	0.98	0.04
69.78	OAE 2	3.4	117	0.90	0.04
72.10	OAE 2	3.0	143	0.87	0.04
74.78	OAE 2	1.4	93.7	1.09	0.04
77.12	OAE 2	2.3	85.2	1.18	0.04
79.15	OAE 2	2.1	141	0.95	0.05
84.07	OAE 2	0.9	153	1.02	0.05
86.48	OAE 2	0.7	121	1.16	0.04
89.23	OAE 2	0.4	46.1	0.91	0.04
90.79	OAE 2	0.5	65.2	0.94	0.04
92.52	OAE 2	0.3	51.3	1.01	0.09
95.18	OAE 2	0.6	132	0.87	0.05
97.34	OAE 2	0.5	105	1.10	0.05
99.62	PCE	2.9	49.4	1.22	0.04
102.17	PCE	0.6	57.0	1.21	0.06
104.41	OAE 2	0.7	103	1.06	0.04
106.83	OAE 2	1.6	67.0	0.94	0.06
109.05	Cenomanian	1.6	79.9	1.03	0.05
111.31	Cenomanian	1.7	98.5	1.07	0.04
113.83	Cenomanian	0.9	70.9	1.07	0.06
115.16	Cenomanian	1.7	60.6	0.90	0.04
117.49	Cenomanian	2.2	59.6	0.93	0.04
122.10	Cenomanian	1.5	43.9	1.01	0.06
126.76	Cenomanian	1.4	49.4	0.92	0.05
128.94	Cenomanian	2.9	76.2	0.83	0.08
131.40	Cenomanian	0.9	33.8	1.11	0.09
133.65	Cenomanian	1.2	52.8	1.01	0.08
139.33	Cenomanian	2.3	65.6	1.04	0.04
147.98	Cenomanian	3.1	54.7	0.97	0.03

Table S1 (continued)

**Depth** m	**Stage**	**Al**[1] wt%	**Ni**[1] ppm	**δ^{60}Ni** ‰	**2 σ** ‰
156.62	Cenomanian	1.7	72.4	0.94	0.06
159.23	Cenomanian	1.5	74.3	1.12	0.05
163.68	Cenomanian	1.5	50.7	0.92	0.07
168.21	Cenomanian	2.4	69.8	0.99	0.05
179.15	Cenomanian	2.7	63.1	0.94	0.03
188.45	Cenomanian	3.6	79.5	1.02	0.08
190.80	Cenomanian	3.1	63.8	0.60	0.08
192.93	Cenomanian	2.7	73.5	0.72	0.07
194.06	Cenomanian	1.5	46.1	0.90	0.09
196.60	Cenomanian	4.1	77.1	0.93	0.08
201.80	MCE	5.0	64.7	0.63	0.07
203.95	MCE	5.5	63.3	0.68	0.08
205.76	MCE	7.5	69.0	1.19	0.07
208.00	MCE	4.5	46.5	0.59	0.07
213.37	MCE	2.1	33.3	0.76	0.08
215.40	MCE	5.0	50.9	0.70	0.07
217.79	Cenomanian	2.6	43.0	0.72	0.08
221.05	Cenomanian	1.3	78.9	0.61	0.06
221.87	Cenomanian	1.7	42.5	0.74	0.07
223.87	Cenomanian	3.0	51.3	0.87	0.07
225.71	Cenomanian	3.4	45.3	0.70	0.07
228.69	Cenomanian	4.4	51.9	0.84	0.05
237.41	Cenomanian	3.5	44.0	0.60	0.09
250.92	Cenomanian	2.8	50.2	0.68	0.07
253.15	Cenomanian	3.1	40.5	0.63	0.08
260.64	Cenomanian	2.4	29.7	0.83	0.09
264.79	Cenomanian	2.0	38.1	0.84	0.07
270.05	Cenomanian	3.9	48.6	0.73	0.05
292.04	Cenomanian	3.4	48.8	0.76	0.06
297.80	Cenomanian	3.6	59.7	0.60	0.06

[1] Data are from Sun et al. *(52)*.

Table S2: The new Ni column chromatography elution steps

	Steps	Regents	Volume Ml
Anion column (AG MP-1M resin)	Cleaning	2% HNO_3	5
	Conditioning	7M $HCl+H_2O_2$	2
	Sample loading	7M $HCl+H_2O_2$	1
	Elute matrix	7M $HCl+H_2O_2$	3×0.1+1.7
Anion column (AG MP-1M resin)	Cleaning	2% HNO_3	1
	Conditioning	0.2M HF	2×0.5
	Sample loading	0.2M HF	1
	Elute matrix	0.2M HF	0.5
Nobias column (Nobias PA-1 resin)	Cleaning	1M HCl	
	Conditioning	MQ H_2O	2
		30mM AcNH4 buffer	2
	Sample loading	0.5M HCl+1M $AcNH_4$ buffer	2+0.2
	Elute matrix	30mM $AcNH_4$ buffer	3×2
		2M NH_4F	4×2.5
		MQ H_2O	2
	Collecting Ni	1M HCl	2

REFERENCES

1. T. W. Lyons, C. T. Reinhard, N. J. Planavsky, The rise of oxygen in Earth's early ocean and atmosphere. *Nature* **506**, 307–315 (2014).

2. C. T. Reinhard, N. J. Planavsky, The history of ocean oxygenation. *Ann. Rev. Mar. Sci.* **14**, 331–353 (2022).

3. H. C. Jenkyns, Geochemistry of oceanic anoxic events. *Geochem. Geophys. Geosyst.* **11**, Q03004 (2010).

4. B. J. Mills, A. J. Krause, I. Jarvis, B. D. Cramer, Evolution of atmospheric O_2 through the Phanerozoic, revisited. *Annu. Rev. Earth Planet. Sci.* **51**, 253–276 (2023).

5. K. Meyer, L. Kump, A. Ridgwell, Biogeochemical controls on photic-zone euxinia during the end-Permian mass extinction. *Geology* **36**, 747–750 (2008).

6. D. P. G. Bond, P. B. Wignall, "Large igneous provinces and mass extinctions: An update" in *Volcanism, Impacts, and Mass Extinctions: Causes and Effects* (Geological Society of America, 2014).

7. B. A. Hoogakker, C. Davis, Y. Wang, S. Kusch, K. Nilsson-Kerr, D. S. Hardisty, A. Jacobel, D. Reyes Macaya, N. Glock, S. Ni, Reviews and syntheses: Review of proxies for low-oxygen paleoceanographic reconstructions. *Biogeosciences* **22**, 863–957 (2025).

8. X. Chen, H.-F. Ling, D. Vance, G. A. Shields-Zhou, M. Zhu, S. W. Poulton, L. M. Och, S.-Y. Jiang, D. Li, L. Cremonese, Rise to modern levels of ocean oxygenation coincided with the Cambrian radiation of animals. *Nat. Commun.* **6**, 7142 (2015).

9. M. O. Clarkson, C. H. Stirling, H. C. Jenkyns, A. J. Dickson, D. Porcelli, C. M. Moy, P. A. Pogge von Strandmann, I. R. Cooke, T. M. Lenton, Uranium isotope evidence for two episodes of deoxygenation during Oceanic Anoxic Event 2. *Proc. Natl. Acad. Sci. U.S.A.* **115**, 2918–2923 (2018).

10. W. Yao, A. Paytan, U. G. Wortmann, Large-scale ocean deoxygenation during the Paleocene-Eocene thermal maximum. *Science* **361**, 804–806 (2018).

11. D. Vance, S. H. Little, C. Archer, V. Cameron, M. B. Andersen, M. J. Rijkenberg, T. W. Lyons, The oceanic budgets of nickel and zinc isotopes: The importance of sulfidic environments as illustrated by the Black Sea. *Philos. Trans. R. Soc. London Ser. A Math. Phys. Eng. Sci.* **374**, 20150294 (2016).

12. E. R. Ciscato, T. R. Bontognali, D. Vance, Nickel and its isotopes in organic-rich sediments: Implications for oceanic budgets and a potential record of ancient seawater. *Earth Planet. Sci. Lett.* **494**, 239–250 (2018).

13. S. H. Little, C. Archer, J. McManus, J. Najorka, A. V. Wegorzewski, D. Vance, Towards balancing the oceanic Ni budget. *Earth Planet. Sci. Lett.* **547**, 116461 (2020).

14. B. Gueguen, O. Rouxel, The nickel isotope composition of the authigenic sink and the diagenetic flux in modern oceans. *Chem. Geol.* **563**, 120050 (2021).

15. Z. He, C. Archer, S. Yang, D. Vance, Sedimentary cycling of zinc and nickel and their isotopes on an upwelling margin: Implications for oceanic budgets and paleoenvironment proxies. *Geochim. Cosmochim. Acta* **343**, 84–97 (2023).

16. S. Fleischmann, J. Du, A. Chatterjee, J. McManus, S. D. Iyer, A. Amonkar, D. Vance, The nickel output to abyssal pelagic manganese oxides: A balanced elemental and isotope budget for the oceans. *Earth Planet. Sci. Lett.* **619**, 118301 (2023).

17. L. E. Wasylenki, R. M. Wells, L. J. Spivak-Birndorf, E. J. Baransky, A. J. Frierdich, Toward mending the marine mass balance model for nickel: Experimentally determined isotope fractionation during Ni sorption to birnessite. *Geochim. Cosmochim. Acta* **379**, 76–88 (2024).

18. X. Bian, S. C. Yang, R. J. Raad, C. E. Odendahl, N. T. Lanning, M. Sieber, K. F. Huang, J. N. Fitzsimmons, T. M. Conway, S. G. John, Distribution and cycling of nickel and nickel isotopes in the Pacific Ocean. *Geophys. Res. Lett.* **51**, e2024GL111115 (2024).

19. L. Chen, A. R. Downey, C. Archer, S. H. Little, W. B. Homoky, C. L. Peacock, Mineralogical controls of the oceanic nickel cycle. *Nat. Commun.* **16**, 7552 (2025).

20. A. J. Dickson, H. C. Jenkyns, D. Porcelli, S. van den Boorn, E. Idiz, Basin-scale controls on the molybdenum-isotope composition of seawater during Oceanic Anoxic Event 2 (Late Cretaceous). *Geochim. Cosmochim. Acta* **178**, 291–306 (2016).

21. C. Siebert, F. Scholz, W. Kuhnt, A new view on the evolution of seawater molybdenum inventories before and during the Cretaceous Oceanic Anoxic Event 2. *Chem. Geol.* **582**, 120399 (2021).

22. K. W. Bruland, Oceanographic distributions of cadmium, zinc, nickel, and copper in the North Pacific. *Earth Planet. Sci. Lett.* **47**, 176–198 (1980).

23. S. W. Ragsdale, Nickel-based enzyme systems. *J. Biol. Chem.* **284**, 18571–18575 (2009).

24. E. D. Goldberg, Marine geochemistry 1. Chemical scavengers of the sea. *J. Geol.* **62**, 249–265 (1954).

25. K. Boström, T. Kraemer, S. Gartner, Provenance and accumulation rates of opaline silica, Al, Ti, Fe, Mn, Cu, Ni and Co in Pacific pelagic sediments. *Chem. Geol.* **11**, 123–148 (1973).

26. P. Böning, T. Shaw, K. Pahnke, H.-J. Brumsack, Nickel as indicator of fresh organic matter in upwelling sediments. *Geochim. Cosmochim. Acta* **162**, 99–108 (2015).

27. J. V. Sorensen, B. Gueguen, B. D. Stewart, J. Peña, O. Rouxel, B. M. Toner, Large nickel isotope fractionation caused by surface complexation reactions with hexagonal birnessite. *Chem. Geol.* **537**, 119481 (2020).

28. T. Sweere, C. Archer, S. Fleischmann, F. Scholz, P. Kraal, D. Vance, Ni isotope cycling in sediments of highly productive upwelling systems. *Earth Planet. Sci. Lett.* **668**, 119549 (2025).

29. N. Lemaitre, J. Du, G. F. de Souza, C. Archer, D. Vance, The essential bioactive role of nickel in the oceans: Evidence from nickel isotopes. *Earth Planet. Sci. Lett.* **584**, 117513 (2022).

30. V. Cameron, D. Vance, Heavy nickel isotope compositions in rivers and the oceans. *Geochim. Cosmochim. Acta* **128**, 195–211 (2014).

31. B. Gueguen, O. Rouxel, Y. Fouquet, Nickel isotopes and rare earth elements systematics in marine hydrogenetic and hydrothermal ferromanganese deposits. *Chem. Geol.* **560**, 119999 (2021).

32. H. Xu, X. Peng, S.-J. Wang, S. Liu, J. Li, H. Yang, Nickel and its isotope in response to differential manganese minerals accumulation in Northwest Pacific sediments. *Mar. Chem.* **273**, 104554 (2025).

33. B. T. Huber, R. D. Norris, K. G. MacLeod, Deep-sea paleotemperature record of extreme warmth during the Cretaceous. *Geology* **30**, 123–126 (2002).

34. Y. Wang, C. Huang, B. Sun, C. Quan, J. Wu, Z. Lin, Paleo-CO_2 variation trends and the Cretaceous greenhouse climate. *Earth Sci. Rev.* **129**, 136–147 (2014).

35. C. L. O'Brien, S. A. Robinson, R. D. Pancost, J. S. S. Damsté, S. Schouten, D. J. Lunt, H. Alsenz, A. Bornemann, C. Bottini, S. C. Brassell, Cretaceous sea-surface temperature evolution: Constraints from TEX86 and planktonic foraminiferal oxygen isotopes. *Earth Sci. Rev.* **172**, 224–247 (2017).

36. S. Beil, W. Kuhnt, A. Holbourn, F. Scholz, J. Oxmann, K. Wallmann, J. Lorenzen, M. Aquit, E. H. Chellai, Cretaceous oceanic anoxic events prolonged by phosphorus cycle feedbacks. *Clim. Past* **16**, 757–782 (2020).

37. S. C. Turgeon, R. A. Creaser, Cretaceous oceanic anoxic event 2 triggered by a massive magmatic episode. *Nature* **454**, 323–326 (2008).

38. H. C. Jenkyns, A. J. Dickson, M. Ruhl, S. H. Van den Boorn, Basalt-seawater interaction, the Plenus Cold Event, enhanced weathering and geochemical change: Deconstructing Oceanic Anoxic Event 2 (Cenomanian–Turonian, Late Cretaceous). *Sedimentology* **64**, 16–43 (2017).

39. L. K. O'Connor, H. C. Jenkyns, S. A. Robinson, S. R. Remmelzwaal, S. J. Batenburg, I. J. Parkinson, A. S. Gale, A re-evaluation of the Plenus Cold Event, and the links between CO_2, temperature, and seawater chemistry during OAE 2. *Paleoceanogr. Paleoclimatol.* **35**, e2019PA003631 (2020).

40. R. Coccioni, S. Galeotti, The mid-Cenomanian Event: Prelude to OAE 2. *Palaeogeogr. Palaeoclimatol. Palaeoecol.* **190**, 427–440 (2003).

41. J. S. Eldrett, C. Ma, S. C. Bergman, B. Lutz, F. J. Gregory, P. Dodsworth, M. Phipps, P. Hardas, D. Minisini, A. Ozkan, An astronomically calibrated stratigraphy of the Cenomanian, Turonian and earliest Coniacian from the Cretaceous Western Interior Seaway, USA: Implications for global chronostratigraphy. *Cretac. Res.* **56**, 316–344 (2015).

42. X. Wang, C. T. Reinhard, N. J. Planavsky, J. D. Owens, T. W. Lyons, T. M. Johnson, Sedimentary chromium isotopic compositions across the Cretaceous OAE2 at Demerara Rise Site 1258. *Chem. Geol.* **429**, 85–92 (2016).

43. T. C. Sweere, A. J. Dickson, H. C. Jenkyns, D. Porcelli, G. M. Henderson, Zinc-and cadmium-isotope evidence for redox-driven perturbations to global micronutrient cycles during Oceanic Anoxic Event 2 (Late Cretaceous). *Earth Planet. Sci. Lett.* **546**, 116427 (2020).

44. C. M. Ostrander, J. D. Owens, S. G. Nielsen, Constraining the rate of oceanic deoxygenation leading up to a Cretaceous Oceanic Anoxic Event (OAE-2: ~94 Ma). *Sci. Adv.* **3**, e1701020 (2017).

45. S. Li, O. Friedrich, S. G. Nielsen, F. Wu, J. D. Owens, Reconciling biogeochemical redox proxies: Tracking variable bottom water oxygenation during OAE-2 using vanadium isotopes. *Earth Planet. Sci. Lett.* **617**, 118237 (2023).

46. S. Floegel, K. Wallmann, C. Poulsen, J. Zhou, A. Oschlies, S. Voigt, W. Kuhnt, Simulating the biogeochemical effects of volcanic CO_2 degassing on the oxygen-state of the deep ocean during the Cenomanian/Turonian Anoxic Event (OAE2). *Earth Planet. Sci. Lett.* **305**, 371–384 (2011).

47. F. M. Monteiro, R. D. Pancost, A. Ridgwell, Y. Donnadieu, Nutrients as the dominant control on the spread of anoxia and euxinia across the Cenomanian-Turonian oceanic anoxic event (OAE2): Model-data comparison. *Paleoceanography* **27**, PA4209 (2012).

48. A. J. Dickson, H. C. Jenkyns, E. Idiz, T. C. Sweere, M. J. Murphy, S. H. van den Boorn, M. Ruhl, J. S. Eldrett, D. Porcelli, New constraints on global geochemical cycling during oceanic anoxic event 2 (late cretaceous) from a 6-million-year long molybdenum-isotope record. *Geochem. Geophys. Geosyst.* **22**, e2020GC009246 (2021).

49. T. Goldberg, S. W. Poulton, T. Wagner, S. F. Kolonic, M. Rehkämper, Molybdenum drawdown during Cretaceous oceanic anoxic event 2. *Earth Planet. Sci. Lett.* **440**, 81–91 (2016).

50. J. D. Owens, B. C. Gill, H. C. Jenkyns, S. M. Bates, S. Severmann, M. M. Kuypers, R. G. Woodfine, T. W. Lyons, Sulfur isotopes track the global extent and dynamics of euxinia during Cretaceous Oceanic Anoxic Event 2. *Proc. Natl. Acad. Sci. U.S.A.* **110**, 18407–18412 (2013).

51. B. S. McDonald, C. A. Partin, B. Sageman, C. Holmden, Uranium isotope reconstruction of ocean deoxygenation during OAE 2 hampered by

uncertainties in fractionation factors and local U-cycling. *Geochim. Cosmochim. Acta* **331**, 143–164 (2022).

52. M. Sun, C. Archer, F. Scholz, T. Sweere, D. Vance, Trace metal evolution of the Late Cretaceous Ocean. *Chem. Geol.* **671**, 122477 (2025).

53. S. Beil, W. Kuhnt, A. E. Holbourn, M. Aquit, S. Flögel, E. H. Chellai, H. Jabour, New insights into Cenomanian paleoceanography and climate evolution from the Tarfaya Basin, southern Morocco. *Cretac. Res.* **84**, 451–473 (2018).

54. F. Scholz, S. Beil, S. Flögel, M. F. Lehmann, A. Holbourn, K. Wallmann, W. Kuhnt, Oxygen minimum zone-type biogeochemical cycling in the Cenomanian-Turonian Proto-North Atlantic across Oceanic anoxic event 2. *Earth Planet. Sci. Lett.* **517**, 50–60 (2019).

55. B. N. Revels, J. Rickli, C. A. Moura, D. Vance, Nickel and its isotopes in the Amazon Basin: The impact of the weathering regime and delivery to the oceans. *Geochim. Cosmochim. Acta* **293**, 344–364 (2021).

56. G. Wu, J.-M. Zhu, X. Wang, T. M. Johnson, Y. He, F. Huang, L.-X. Wang, S.-C. Lai, Nickel isotopic composition of the upper continental crust. *Geochim. Cosmochim. Acta* **332**, 263–284 (2022).

57. P. Böning, H. Fröllje, M. Beck, B. Schnetger, H.-J. Brumsack, Underestimation of the authigenic fraction of Cu and Ni in organic-rich sediments. *Mar. Geol.* **323**, 24–28 (2012).

58. P. Böning, H.-J. Brumsack, M. E. Böttcher, B. Schnetger, C. Kriete, J. Kallmeyer, S. L. Borchers, Geochemistry of Peruvian near-surface sediments. *Geochim. Cosmochim. Acta* **68**, 4429–4451 (2004).

59. T. Sweere, S. van den Boorn, A. J. Dickson, G.-J. Reichart, Definition of new trace-metal proxies for the controls on organic matter enrichment in marine sediments based on Mn, Co, Mo and Cd concentrations. *Chem. Geol.* **441**, 235–245 (2016).

60. F. Scholz, Identifying oxygen minimum zone-type biogeochemical cycling in Earth history using inorganic geochemical proxies. *Earth Sci. Rev.* **184**, 29–45 (2018).

61. H. Schunck, G. Lavik, D. K. Desai, T. Großkopf, T. Kalvelage, C. R. Löscher, A. Paulmier, S. Contreras, H. Siegel, M. Holtappels, Giant hydrogen sulfide plume in the oxygen minimum zone off Peru supports chemolithoautotrophy. *PLOS ONE* **8**, e68661 (2013).

62. W. Kuhnt, A. E. Holbourn, S. Beil, M. Aquit, T. Krawczyk, S. Flögel, E. H. Chellai, H. Jabour, Unraveling the onset of Cretaceous Oceanic Anoxic Event 2 in an extended sediment archive from the Tarfaya-Laayoune Basin, Morocco. *Paleoceanography* **32**, 923–946 (2017).

63. P. A. Pogge von Strandmann, H. C. Jenkyns, R. G. Woodfine, Lithium isotope evidence for enhanced weathering during Oceanic Anoxic Event 2. *Nat. Geosci.* **6**, 668–672 (2013).

64. M. Dellinger, J. Gaillardet, J. Bouchez, D. Calmels, P. Louvat, A. Dosseto, C. Gorge, L. Alanoca, L. Maurice, Riverine Li isotope fractionation in the Amazon River basin controlled by the weathering regimes. *Geochim. Cosmochim. Acta* **164**, 71–93 (2015).

65. B. N. Revels, J. Rickli, C. A. Moura, D. Vance, The riverine flux of molybdenum and its isotopes to the ocean: Weathering processes and dissolved-particulate partitioning in the Amazon basin. *Earth Planet. Sci. Lett.* **559**, 116773 (2021).

66. T. F. Nägler, A. D. Anbar, C. Archer, T. Goldberg, G. W. Gordon, N. D. Greber, C. Siebert, Y. Sohrin, D. Vance, Proposal for an international molybdenum isotope measurement standard and data representation. *Geostand. Geoanal. Res.* **38**, 149–151 (2014).

67. N. Estrade, C. Cloquet, G. Echevarria, T. Sterckeman, T. Deng, Y. Tang, J.-L. Morel, Weathering and vegetation controls on nickel isotope fractionation in surface ultramafic environments (Albania). *Earth Planet. Sci. Lett.* **423**, 24–35 (2015).

68. G. Ratié, D. Jouvin, J. Garnier, O. Rouxel, S. Miska, E. Guimarães, L. C. Vieira, Y. Sivry, I. Zelano, E. Montarges-Pelletier, Nickel isotope fractionation during tropical weathering of ultramafic rocks. *Chem. Geol.* **402**, 68–76 (2015).

69. G. L. Arnold, A. D. Anbar, J. Barling, T. Lyons, Molybdenum isotope evidence for widespread anoxia in mid-Proterozoic oceans. *Science* **304**, 87–90 (2004).

70. B. Kendall, G. W. Gordon, S. W. Poulton, A. D. Anbar, Molybdenum isotope constraints on the extent of late Paleoproterozoic ocean euxinia. *Earth Planet. Sci. Lett.* **307**, 450–460 (2011).

71. A. J. Dickson, A molybdenum-isotope perspective on Phanerozoic deoxygenation events. *Nat. Geosci.* **10**, 721–726 (2017).

72. G. Zhang, Y. Zhu, Y. Deng, J. Cao, P. Wang, A. Yang, G. He, B. Zhao, M. Zhao, Balancing the oceanic Zn isotope budget: The key role of deep-sea pelagic sediments. *Geology* **52**, 789–793 (2024).

73. J. K. Caves, A. B. Jost, K. V. Lau, K. Maher, Cenozoic carbon cycle imbalances and a variable weathering feedback. *Earth Planet. Sci. Lett.* **450**, 152–163 (2016).

74. S. Misra, P. N. Froelich, Lithium isotope history of Cenozoic seawater: Changes in silicate weathering and reverse weathering. *Science* **335**, 818–823 (2012).

75. M. Sun, C. Archer, F. Scholz, T. Sweere, D. Vance, in *Goldschmidt 2025 Conference* (GOLDSCHMIDT, 2025).

76. R. A. Berner, D. E. Canfield, A new model for atmospheric oxygen over Phanerozoic time. *Am. J. Sci.* **289**, 333–361 (1989).

77. J. M. Hayes, H. Strauss, A. J. Kaufman, The abundance of ^{13}C in marine organic matter and isotopic fractionation in the global biogeochemical cycle of carbon during the past 800 Ma. *Chem. Geol.* **161**, 103–125 (1999).

78. K. W. Hansen, K. Wallmann, Cretaceous and Cenozoic evolution of seawater composition, atmospheric O_2 and CO_2: A model perspective. *Am. J. Sci.* **303**, 94–148 (2003).

79. P. G. Falkowski, M. E. Katz, A. J. Milligan, K. Fennel, B. S. Cramer, M. P. Aubry, R. A. Berner, M. J. Novacek, W. M. Zapol, The rise of oxygen over the past 205 million years and the evolution of large placental mammals. *Science* **309**, 2202–2204 (2005).

80. I. J. Glasspool, A. C. Scott, Phanerozoic concentrations of atmospheric oxygen reconstructed from sedimentary charcoal. *Nat. Geosci.* **3**, 627–630 (2010).

81. B. J. Mills, C. M. Belcher, T. M. Lenton, R. J. Newton, A modeling case for high atmospheric oxygen concentrations during the Mesozoic and Cenozoic. *Geology* **44**, 1023–1026 (2016).

82. S. M. Newby, S. A. Young, T. R. Them, B. C. Gill, J. D. Owens, Balancing redox budgets: Mechanisms for prolonging anoxia during major carbon burial events. *Am. J. Sci.* **325**, 12 (2025).

83. R. E. Martin, Cyclic and secular variation in microfossil biomineralization: Clues to the biogeochemical evolution of Phanerozoic oceans. *Glob. Planet. Change* **11**, 1–23 (1995).

84. P. G. Falkowski, M. E. Katz, A. H. Knoll, A. Quigg, J. A. Raven, O. Schofield, F. Taylor, The evolution of modern eukaryotic phytoplankton. *Science* **305**, 354–360 (2004).

85. M. E. Katz, Z. V. Finkel, D. Grzebyk, A. H. Knoll, P. G. Falkowski, Evolutionary trajectories and biogeochemical impacts of marine eukaryotic phytoplankton. *Annu. Rev. Ecol. Evol. Syst.* **35**, 523–556 (2004).

86. E. V. Armbrust, The life of diatoms in the world's oceans. *Nature* **459**, 185–192 (2009).

87. T. M. Lenton, R. A. Boyle, S. W. Poulton, G. A. Shields-Zhou, N. J. Butterfield, Co-evolution of eukaryotes and ocean oxygenation in the Neoproterozoic era. *Nat. Geosci.* **7**, 257–265 (2014).

88. W. Lu, A. Ridgwell, E. Thomas, D. S. Hardisty, G. Luo, T. J. Algeo, M. R. Saltzman, B. C. Gill, Y. Shen, H.-F. Ling, Late inception of a resiliently oxygenated upper ocean. *Science* **361**, 174–177 (2018).

89. T. D. Frank, M. A. Arthur, Tectonic forcings of Maastrichtian ocean-climate evolution. *Paleoceanography* **14**, 103–117 (1999).

90. S. A. Robinson, D. Vance, Widespread and synchronous change in deep-ocean circulation in the North and South Atlantic during the Late Cretaceous. *Paleoceanography* **27**, PA1102 (2012).

91. W. Dummann, P. Hofmann, J. O. Herrle, M. Frank, T. Wagner, The early opening of the Equatorial Atlantic gateway and the evolution of Cretaceous peak warming. *Geology* **51**, 476–480 (2023).

92. K. MacLeod, C. Isaza Londoño, E. Martin, Á. Jiménez Berrocoso, C. Basak, Changes in North Atlantic circulation at the end of the Cretaceous greenhouse interval. *Nat. Geosci.* **4**, 779–782 (2011).

93. M. Sun, C. Archer, D. Vance, New methods for the chemical isolation and stable isotope measurement of multiple transition metals, with application to the Earth sciences. *Geostand. Geoanal. Res.* **45**, 643–658 (2021).

94. C. N. Maréchal, P. Télouk, F. Albarède, Precise analysis of copper and zinc isotopic compositions by plasma-source mass spectrometry. *Chem. Geol.* **156**, 251–273 (1999).

95. J. Faris, Adsorption of elements from hydrofluoric acid by anion exchange. *Anal. Chem.* **32**, 520–522 (1960).

96. S. Takano, M. Tsuchiya, S. Imai, Y. Yamamoto, Y. Fukami, K. Suzuki, Y. Sohrin, Isotopic analysis of nickel, copper, and zinc in various freshwater samples for source identification. *Geochem. J.* **55**, 171–183 (2021).

97. C. Archer, D. Vance, The isotopic signature of the global riverine molybdenum flux and anoxia in the ancient oceans. *Nat. Geosci.* **1**, 597–600 (2008).

98. B. Kendall, T. W. Dahl, A. D. Anbar, The stable isotope geochemistry of molybdenum. *Rev. Mineral. Geochem.* **82**, 683–732 (2017).

99. T. J. Algeo, C. Li, Redox classification and calibration of redox thresholds in sedimentary systems. *Geochim. Cosmochim. Acta* **287**, 8–26 (2020).

100. T. W. Dahl, E. U. Hammarlund, A. D. Anbar, D. P. Bond, B. C. Gill, G. W. Gordon, A. H. Knoll, A. T. Nielsen, N. H. Schovsbo, D. E. Canfield, Devonian rise in atmospheric oxygen correlated to the radiations of terrestrial plants and large predatory fish. *Proc. Natl. Acad. Sci. U.S.A.* **107**, 17911–17915 (2010).

101. K.-C. Emeis, J. K. Whelan, M. Tarafa, Sedimentary and geochemical expressions of oxic and anoxic conditions on the Peru shelf. *Geol. Soc. Lond. Spec. Publ.* **58**, 155–170 (1991).

102. C. T. Reinhard, N. J. Planavsky, L. J. Robbins, C. A. Partin, B. C. Gill, S. V. Lalonde, A. Bekker, K. O. Konhauser, T. W. Lyons, Proterozoic ocean redox and biogeochemical stasis. *Proc. Natl. Acad. Sci. U.S.A.* **110**, 5357–5362 (2013).

103. F. Scholz, C. Siebert, A. W. Dale, M. Frank, Intense molybdenum accumulation in sediments underneath a nitrogenous water column and

implications for the reconstruction of paleo-redox conditions based on molybdenum isotopes. *Geochim. Cosmochim. Acta* **213**, 400–417 (2017).

104. C. Scott, T. Lyons, A. Bekker, Y.-a. Shen, S. Poulton, X.-l. Chu, A. Anbar, Tracing the stepwise oxygenation of the Proterozoic ocean. *Nature* **452**, 456–459 (2008).

105. R. L. Poulson Brucker, J. McManus, S. Severmann, W. M. Berelson, Molybdenum behavior during early diagenesis: Insights from Mo isotopes. *Geochem. Geophys. Geosyst.* **10**, Q06010 (2009).

106. S. Fleischmann, F. Scholz, J. Du, J. Scholten, D. Vance, Processes controlling nickel and its isotopes in anoxic sediments of a seasonally hypoxic bay. *Geochim. Cosmochim. Acta* **391**, 1–15 (2025).

107. J. L. Morford, S. Emerson, The geochemistry of redox sensitive trace metals in sediments. *Geochim. Cosmochim. Acta* **63**, 1735–1750 (1999).

108. M.-E. Carr, Estimation of potential productivity in Eastern Boundary Currents using remote sensing. *Deep Sea Res. 2 Top. Stud. Oceanogr.* **49**, 59–80 (2001).

109. D. E. LaRowe, S. Arndt, J. A. Bradley, E. Burwicz, A. W. Dale, J. P. Amend, Organic carbon and microbial activity in marine sediments on a global scale throughout the Quaternary. *Geochim. Cosmochim. Acta* **286**, 227–247 (2020).

110. R. A. Jahnke, "Global synthesis[1]" in *Carbon and Nutrient Fluxes in Continental Margins: A Global Synthesis* (Springer, 2010), pp. 597–615.

111. B. B. Jørgensen, F. Wenzhöfer, M. Egger, R. N. Glud, Sediment oxygen consumption: Role in the global marine carbon cycle. *Earth Sci. Rev.* **228**, 103987 (2022).

112. F. Gäng, P. Böning, V. Brüchert, N. Lahajnar, K. Pahnke, Critical assessment of U, Ba and Ni as redox and productivity proxies in organic-rich sediments underneath dynamic, highly productive waters. *Geochim. Cosmochim. Acta* **348**, 206–220 (2023).

113. J. I. Hedges, R. G. Keil, Sedimentary organic matter preservation: An assessment and speculative synthesis. *Mar. Chem.* **49**, 81–115 (1995).

114. L. A. Tegler, T. J. Horner, V. Galy, S. M. Bent, Y. Wang, H. H. Kim, Ö. Z. Mete, S. G. Nielsen, Distribution and drivers of organic carbon sedimentation along the continental margins. *AGU Adv.* **5**, e2023AV001000 (2024).

115. S. Borchers, B. Schnetger, P. Böning, H. J. Brumsack, Geochemical signatures of the Namibian diatom belt: Perennial upwelling and intermittent anoxia. *Geochem. Geophys. Geosyst.* **6**, Q06006 (2005).

116. J. McManus, W. M. Berelson, S. Severmann, R. L. Poulson, D. E. Hammond, G. P. Klinkhammer, C. Holm, Molybdenum and uranium geochemistry in continental margin sediments: Paleoproxy potential. *Geochim. Cosmochim. Acta* **70**, 4643–4662 (2006).

117. F. Scholz, C. Hensen, A. Noffke, A. Rohde, V. Liebetrau, K. Wallmann, Early diagenesis of redox-sensitive trace metals in the Peru upwelling area–

response to ENSO-related oxygen fluctuations in the water column. *Geochim. Cosmochim. Acta* **75**, 7257–7276 (2011).

118. S. H. Little, D. Vance, T. W. Lyons, J. McManus, Controls on trace metal authigenic enrichment in reducing sediments: Insights from modern oxygen-deficient settings. *Am. J. Sci.* **315**, 77–119 (2015).

119. R. Salvatteci, D. Gutiérrez, A. Sifeddine, L. Ortlieb, E. Druffel, M. Boussafir, R. Schneider, Centennial to millennial-scale changes in oxygenation and productivity in the Eastern Tropical South Pacific during the last 25,000 years. *Quat. Sci. Rev.* **131**, 102–117 (2016).

120. M. L. Abshire, J. D. Owens, J. Cofrancesco, M. Inthorn, N. Riedinger, Geochemical signatures of redepositional environments: The Namibian continental margin. *Mar. Geol.* **429**, 106316 (2020).

121. A. Plass, A. W. Dale, F. Scholz, Sedimentary cycling and benthic fluxes of manganese, cobalt, nickel, copper, zinc and cadmium in the Peruvian oxygen minimum zone. *Mar. Chem.* **233**, 103982 (2021).

122. M. Kossack, F. Scholz, C. H. Anderson, P. Vosteen, C.-C. Su, V. Mohrholz, M. Zabel, Sedimentary molybdenum and uranium cycling under seasonally contrasting redox conditions on the Namibian Shelf. *Geochim. Cosmochim. Acta* **358**, 174–191 (2023).

123. H.-J. Brumsack, Geochemistry of recent TOC-rich sediments from the Gulf of California and the Black Sea. *Geol. Rdsch.* **78**, 851–882 (1989).

124. P. Böning, S. Cuypers, M. Grunwald, B. Schnetger, H.-J. Brumsack, Geochemical characteristics of Chilean upwelling sediments at~ 36° S. *Mar. Geol.* **220**, 1–21 (2005).

125. P. Böning, H.-J. Brumsack, B. Schnetger, M. Grunwald, Trace element signatures of Chilean upwelling sediments at ~36°S. *Mar. Geol.* **259**, 112–121 (2009).

126. C. H. van der Weijden, G.-J. Reichart, B. J. van Os, Sedimentary trace element records over the last 200 kyr from within and below the northern Arabian Sea oxygen minimum zone. *Mar. Geol.* **231**, 69–88 (2006).

127. D. Z. Piper, W. E. Dean, *Trace-Element Deposition in the Cariaco Basin, Venezuela Shelf, Under Sulfate-Reducing Conditions: A History of the Local Hydrography and Global Climate, 20 ka to the Present* (US Government Printing Office, 2002).

128. A. Brüske, S. Weyer, M.-Y. Zhao, N. Planavsky, A. Wegwerth, N. Neubert, O. Dellwig, K. Lau, T. Lyons, Correlated molybdenum and uranium isotope signatures in modern anoxic sediments: Implications for their use as paleo-redox proxy. *Geochim. Cosmochim. Acta* **270**, 449–474 (2020).

129. H. Lüschen, "Vergleichende anorganisch-geochemische Untersuchungen an phanerozoischen C org-reichen Sedimenten: ein Beitrag zur Charakterisierung ihrer Fazies," thesis, Universität Oldenburg, Oldenburg, Germany (2004).

130. T. Fujii, F. Moynier, N. Dauphas, M. Abe, Theoretical and experimental investigation of nickel isotopic fractionation in species relevant to modern and ancient oceans. *Geochim. Cosmochim. Acta* **75**, 469–482 (2011).

131. B. E. Erickson, G. R. Helz, Molybdenum (VI) speciation in sulfidic waters: Stability and lability of thiomolybdates. *Geochim. Cosmochim. Acta* **64**, 1149–1158 (2000).

132. T. J. Algeo, T. W. Lyons, Mo–total organic carbon covariation in modern anoxic marine environments: Implications for analysis of paleoredox and paleohydrographic conditions. *Paleoceanography* **21**, PA1016 (2006).

133. P. L. Fraga-Ferreira, C. Siebert, M. Frank, F. Scholz, Diagenetic and hydrothermal processes produce heavy molybdenum isotope signatures in pelagic sediments of the North and South Pacific. *Geochem. Geophys. Geosyst.* **27**, e2025GC012749 (2026).

134. M. L. Bender, T.-L. Ku, W. S. Broecker, Accumulation rates of manganese in pelagic sediments and nodules. *Earth Planet. Sci. Lett.* **8**, 143–148 (1970).

135. G. Shimmield, N. Price, The behaviour of molybdenum and manganese during early sediment diagenesis—Offshore Baja California, Mexico. *Mar. Chem.* **19**, 261–280 (1986).

136. C. Siebert, T. F. Nägler, F. von Blanckenburg, J. D. Kramers, Molybdenum isotope records as a potential new proxy for paleoceanography. *Earth Planet. Sci. Lett.* **211**, 159–171 (2003).

137. F. Scholz, J. McManus, S. Sommer, The manganese and iron shuttle in a modern euxinic basin and implications for molybdenum cycling at euxinic ocean margins. *Chem. Geol.* **355**, 56–68 (2013).

138. R. L. Rudnick, S. Gao, “4.1—Composition of the continental crust” in *Treatise on Geochemistry*, H. D. Holland, K. K. Turekian, Eds. (Elsevier, ed. 2, 2014), pp. 1–51.

139. C. L. Peacock, Physiochemical controls on the crystal-chemistry of Ni in birnessite: Genetic implications for ferromanganese precipitates. *Geochim. Cosmochim. Acta* **73**, 3568–3578 (2009).

140. K. Von Damm, J. M. Edmond, B. Grant, C. Measures, B. Walden, R. Weiss, Chemistry of submarine hydrothermal solutions at 21°N, East Pacific Rise. *Geochim. Cosmochim. Acta* **49**, 2197–2220 (1985).

141. M. Van Hulten, R. Middag, J.-C. Dutay, H. De Baar, M. Roy-Barman, M. Gehlen, A. Tagliabue, A. Sterl, Manganese in the west Atlantic Ocean in the context of the first global ocean circulation model of manganese. *Biogeosciences* **14**, 1123–1152 (2017).

142. F. T. Manheim, C. M. Lane-Bostwick, Chemical composition of ferromanganese crusts in the world ocean: A review and comprehensive database (Open-File Report, U.S. Geological Survey, 1989).

143. S. Krishnaswami, Authigenic transition elements in Pacific pelagic clays. *Geochim. Cosmochim. Acta* **40**, 425–434 (1976).

144. S. Calvert, N. Price, Geochemical variation in ferromanganese nodules and associated sediments from the Pacific Ocean. *Mar. Chem.* **5**, 43–74 (1977).

145. S. Calvert, D. Piper, Geochemistry of ferromanganese nodules from DOMES Site A, Northern Equatorial Pacific: Multiple diagenetic metal sources in the deep sea. *Geochim. Cosmochim. Acta* **48**, 1913–1928 (1984).

146. C. Heller, T. Kuhn, G. J. Versteegh, A. V. Wegorzewski, S. Kasten, The geochemical behavior of metals during early diagenetic alteration of buried manganese nodules. *Deep Sea Res. 1 Oceanogr. Res. Pap.* **142**, 16–33 (2018).

147. K. Boström, M. Peterson, O. Joensuu, D. E. Fisher, Aluminum-poor ferromanganoan sediments on active oceanic ridges. *J. Geophys. Res.* **74**, 3261–3270 (1969).

148. S. Moorby, D. Cronan, The geochemistry of hydrothermal and pelagic sediments from the Galapagos Hydrothermal Mounds Field, DSDP Leg 70. *Mineral. Mag.* **47**, 291–300 (1983).

149. J. R. Hein, M. S. Schulz, R. E. Dunham, R. J. Stern, S. H. Bloomer, Diffuse flow hydrothermal manganese mineralization along the active Mariana and southern Izu-Bonin arc system, western Pacific. *J. Geophys. Res. Solid Earth* **113**, B08S14 (2008).

150. Z. Zeng, H. Ouyang, X. Yin, S. Chen, X. Wang, L. Wu, Formation of Fe–Si–Mn oxyhydroxides at the PACMANUS hydrothermal field, Eastern Manus

Basin: Mineralogical and geochemical evidence. *J. Asian Earth Sci.* **60**, 130–146 (2012).

151. A. Pan, Q. Yang, H. Zhou, F. Ji, H. Wang, R. D. Pancost, Geochemical impacts of hydrothermal activity on surface deposits at the Southwest Indian Ridge. *Deep Sea Res. 1 Oceanogr. Res. Pap.* **139**, 1–13 (2018).

152. K. T. Goto, Y. Sekine, G. Shimoda, J. R. Hein, S. Aoki, A. Ishikawa, K. Suzuki, G. W. Gordon, A. D. Anbar, A framework for understanding Mo isotope records of Archean and Paleoproterozoic Fe-and Mn-rich sedimentary rocks: Insights from modern marine hydrothermal Fe-Mn oxides. *Geochim. Cosmochim. Acta* **280**, 221–236 (2020).

153. Z. Sun, H. Zhou, Q. Yang, Z. Sun, S. Bao, H. Yao, Hydrothermal Fe–Si–Mn oxide deposits from the Central and South Valu Fa Ridge, Lau Basin. *Appl. Geochem.* **26**, 1192–1204 (2011).

154. J.-M. Lee, M. I. Heller, P. J. Lam, Size distribution of particulate trace elements in the US GEOTRACES Eastern Pacific Zonal Transect (GP16). *Mar. Chem.* **201**, 108–123 (2018).

155. T. Bralower, H. Thierstein, Organic carbon and metal accumulation rates in Holocene and mid-Cretaceous sediments: Palaeoceanographic significance. *Geol. Soc. Lond. Spec. Publ.* **26**, 345–369 (1987).

156. R. Francois, A study on the regulation of the concentrations of some trace metals (Rb, Sr, Zn, Pb, Cu, V, Cr, Ni, Mn and Mo) in Saanich Inlet sediments, British Columbia, Canada. *Mar. Geol.* **83**, 285–308 (1988).

157. D. Z. Piper, S. Calvert, A marine biogeochemical perspective on black shale deposition. *Earth Sci. Rev.* **95**, 63–96 (2009).

158. B. Somayajulu, G. Heath, T. Moore Jr., D. Cronan, Rates of accumulation of manganese nodules and associated sediment from the equatorial Pacific. *Geochim. Cosmochim. Acta* **35**, 621–624 (1971).

159. K. K. Bertine, K. K. Turekian, Molybdenum in marine deposits. *Geochim. Cosmochim. Acta* **37**, 1415–1434 (1973).

160. M. Leinen, D. Stakes, Metal accumulation rates in the central equatorial Pacific during Cenozoic time. *Geol. Soc. Am. Bull.* **90**, 357–375 (1979).

161. J. Thomson, M. Carpenter, S. Colley, T. Wilson, H. Elderfield, H. Kennedy, Metal accumulation rates in northwest Atlantic pelagic sediments. *Geochim. Cosmochim. Acta* **48**, 1935–1948 (1984).
